# Discovery of pyrrole derivatives as acetylcholinesterase-sparing butyrylcholinesterase inhibitor

**DOI:** 10.3389/fphar.2022.1043397

**Published:** 2022-12-06

**Authors:** Shouyuan Sun, Tao Shi, Yan Peng, Honghua Zhang, Linsheng Zhuo, Xue Peng, Qien Li, Manxia Wang, Shuzhi Wang, Zhen Wang

**Affiliations:** ^1^ Lanzhou University Second Hospital, Lanzhou, China; ^2^ School of Pharmacy, Lanzhou University, Lanzhou, China; ^3^ School of Pharmaceutical Science, Hengyang Medical School, University of South China, Hengyang, China; ^4^ Tibetan Medical College, Qinghai University, Xining, China

**Keywords:** substituted pyrroles, acetylcholinesterase, butyrylcholinesterase, molecular docking, enzyme kinetic

## Abstract

Inspired by the crucial roles of (hetero)aryl rings in cholinesterase inhibitors and the pyrrole ring in new drug discovery, we synthesized 19 pyrrole derivatives and investigated their cholinesterase inhibitory activity. As a result, compounds 3o, 3p, and 3s with a 1,3-diaryl-pyrrole skeleton showed high selectivity toward BChE over AChE with a best IC_50_ value of 1.71 ± 0.087 µM, which were comparable to donepezil. The pharmaceutical potential of these structures was further predicted and compounds 3o and 3p were proved to meet well with the Lipinsky’s five rules. In combination of the inhibition kinetic studies with the results of molecular docking, we concluded that compound 3p inhibited BChE in a mixed competitive mode. This research has proved the potential of the 1,3-diaryl-pyrrole skeleton as a kind of selective BChE inhibitor.

## 1 Introduction

It is reported that there are mainly two categories of cholinesterase in vertebrates, including acetylcholinesterase (AChE) and butyrylcholinesterase (BChE), and they are mainly distributed in the brain and blood, respectively (Garibov et al., 2016; Burmaoglu et al., 2019; Erdemir et al., 2019). In terms of the function, they could hydrolyze acetylcholine to terminate impulse transmission at cholinergic synapses, thus preventing the restoration of cholinergic neurons and leading to cognitive impairment (Pohanka, 2011; Cavallaro et al., 2018). Therefore, cholinesterase inhibition is an effective therapeutic approach for the treatment of a variety of cholinesterase-related diseases, such as Alzheimer’s disease (AD), glaucoma, or to antagonize muscle relaxation (Cavallaro et al., 2018). It is reported that the expression level of these two types of cholinesterases are dynamic with the progress of AD, and thus the selective inhibition on AChE or BChE may be effective for AD in different stages. However, due to the high homogeneity of AChE and BChE in the amino acid sequence, the development of selective cholinesterase inhibitors (ChEIs) is difficult and side effects are always emerged in clinical practice (Jing et al., 2019; Mishra et al., 2019; Zhou et al., 2019). Therefore, it is of crucial importance to develop selective cholinesterase inhibitors (ChEIs).

**FIGURE 1:**
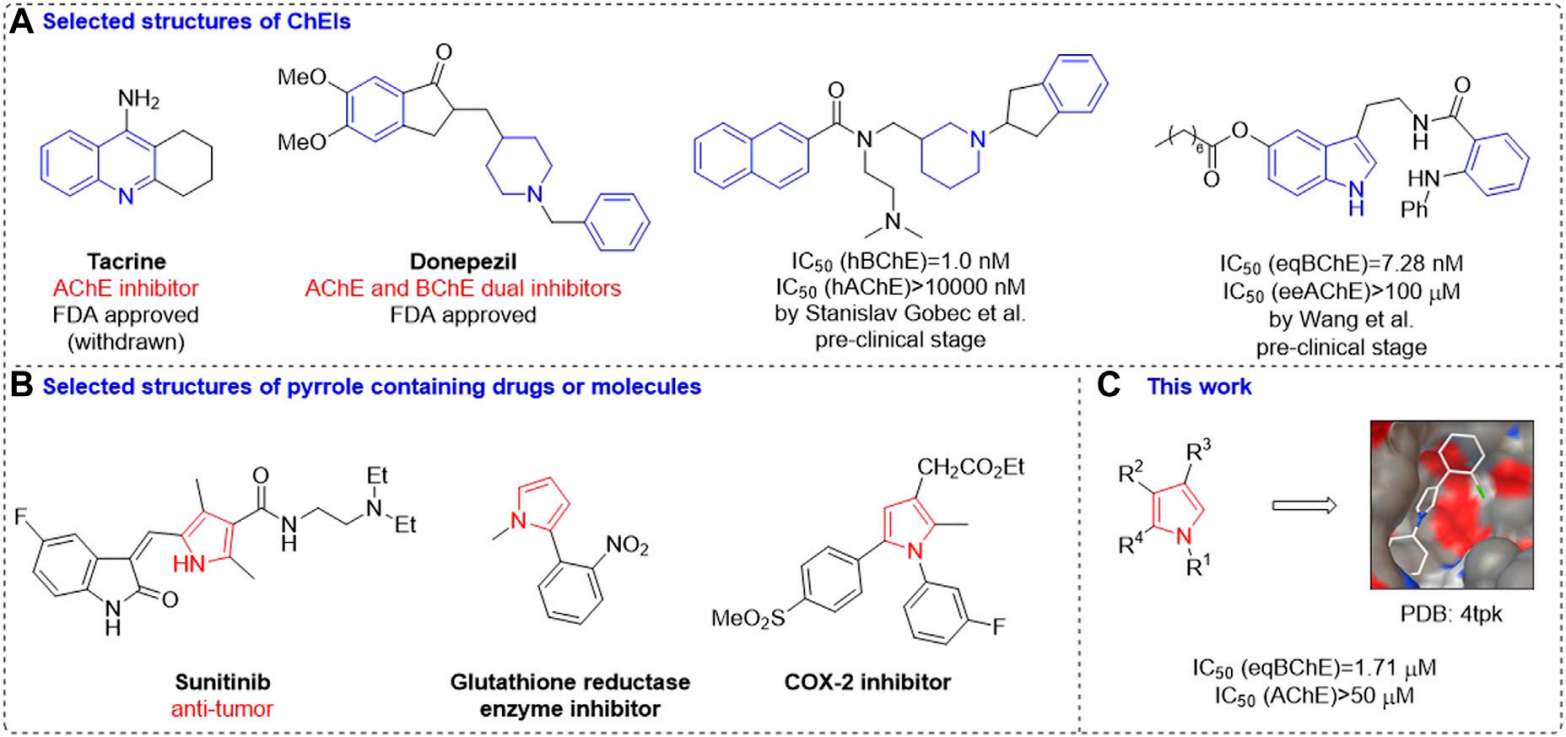
Selected structures of ChEIs **(A)**; Selected structures of pyrrole containing drugs or molecules **(B)**; This work **(C)**.

Up to now, Food and Drug Administration (FDA) has approved several ChEIs for treating AD. (Zhou et al., 2019; Du et al., 2018; Sanabria-Castro et al., 2017; Li et al., 2018; Contestabile, 2011) Among them, most of them have selective inhibition on AChE or dual inhibition on both AChE and BuChE (Lilienfeld, 2002; Colovic et al., 2013; Telpoukhovskaia et al., 2016). As far as we could know, there is no selective BChE inhibitors being approved by FDA, although the discovery of small molecules with selective BChE inhibitory activities is continually increasing in recent years (Jing et al., 2019; Zhang et al., 2022). Taking deep insight into the structures of these approved drugs and potential molecules, it could be found that (hetero)aryl rings especially nitrogen heterocycles (Kumari et al., 2022) are the most prevalent structural motifs (Figure 1A). In view of the important function of the pyrrole skeleton in drugdiscovery, (Javahershenas et al., 2020), and biological activity of pyrrole derivatives for different enzymes (Alp et al., 2010; Kocaoğlu et al., 2019; Obaid et al., 2022) (Figure 1B) and to further expand the chemical space of ChEIs, we synthesized a series of pyrrole derivatives and investigated their cholinesterase inhibitory activity as well as structural basis to BChE (Figure 1C).

## 2 Results and discussion

### 2.1 Chemistry

**SCHEME 1:**
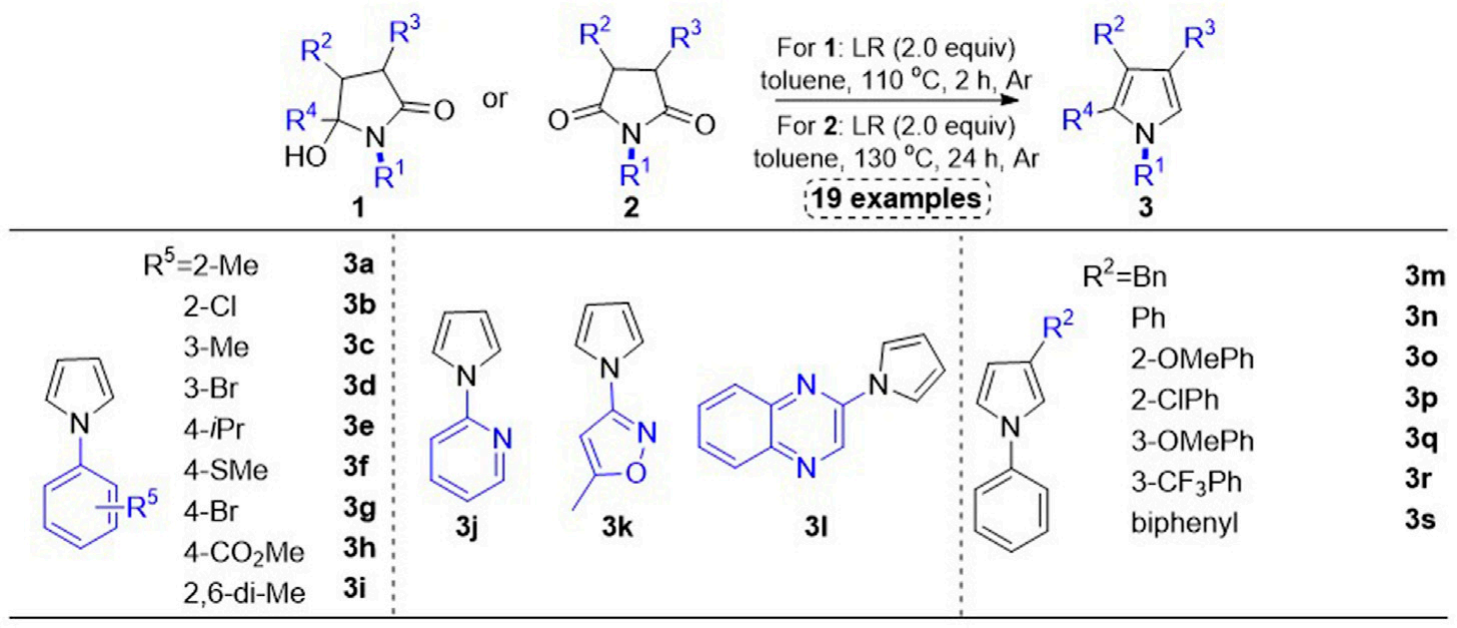
Synthetic route to N-phenyl substituted pyrroles. Method 1: Lawesson’s reagent (2.0 equiv), toluene (0.1 M), 110°C, 2 h, Ar. Method B: Lawesson’s reagent (2.0 equiv), toluene (0.1 M), 130°C, 24 h, Ar.

In this work, we disclosed the syntheses of 19 substituted pyrroles, which were used to explore the influence of electronic effects and steric hindrance of substituents at the five-membered pyrrole ring on the cholinesterase inhibition. As for the synthetic route to these compounds, Lawesson’s reagent promoted deoxygenation of *γ*-hydroxylactams 1 or succinimides 2 realized the one-step syntheses of pyrroles 3 in moderate to good yields, which has been reported in our previously published works (Scheme 1 (Shi et al., 2022).

### 2.2 Biological evaluation

#### 2.2.1 Determination of cholinesterase inhibitory activity and structure-activity relationships

The cholinesterase inhibitory activity of synthesized molecules was assessed by using the most frequently employed Ellman’s method (Ellman et al., 1961; Arslan et al., 2019; Durdagi et al., 2019; Özil et al., 2019; Salehi et al., 2019; Arslan et al., 2020; Gülçin et al., 2021; Gümüş et al., 2022; Onder et al., 2022; Zhang et al., 2022). Under a frequently employed incubation time of 25 min, all the synthesized compounds showed no inhibition on AChE (IC_50_ > 50 µM). With regarding to the BChE inhibitory activity, only compounds 3o, 3p, and 3s have inhibition on BChE, and their IC_50_ values were 5.37 ± 0.36, 1.71 ± 0.087, and 3.76 ± 0.25 µM, respectively, which were comparable to the positive drug (Table 1). The IC_50_ values of donepezil against AChE and BChE were close to the literature data (*ee*AChE: 2.45 ± 0.08 µM^16^; 0.25 ± 0.42 µM^31^; 0.023 ± 0.0013 µM^32^; *eq*BChE: 3.70 ± 0.28 µM^16^; 2.66 ± 0.79 µM^31^; 3.4 ± 0.023 µM^32^;). This result implied that (hetero)aryl substituents on the nitrogen atom have no beneficial influence on the cholinesterase inhibitory activity.

**TABLE 1:** IC_50_ (µM) values of compounds **3a**-**3s** on *eq*BChE and *ee*AChE*^a^*.

Compound	IC_50_ *eq*BChE	IC_50_ *ee*AChE	Compound	IC_50_ *eq*BChE	IC_50_ *ee*AChE
**3a**	>50	>50	**3k**	>50	>50
**3b**	>50	>50	**3l**	>50	>50
**3c**	>50	>50	**3m**	>50	>50
**3d**	>50	>50	**3n**	>50	>50
**3e**	>50	>50	**3o**	5.37 ± 0.36	>50
**3f**	>50	>50	**3p**	1.71 ± 0.087	>50
**3g**	>50	>50	**3q**	>50	>50
**3h**	>50	>50	**3r**	>50	>50
**3i**	>50	>50	**3s**	3.76 ± 0.25	>50
**3j**	>50	>50	**Donepezil**	1.58 ± 0.066	7.94 ± 0.99

^a^ AChE was from the electric eel, and BChE was from equine serum. The results were expressed as mean ± SD, of at least three independent experiments.

Bold values mean the compound number.

#### 2.2.2 Prediction of drug-like properties

After proving the *eq*BChE inhibitory effects of compounds 3o, 3p, and 3s, we then predicted the druggability of the above molecules by using the SwissADME and the Molinspiration calculation software (Table 2). As a result, the molecular weights of all compounds are below 500 Da. The number of hydrogen bond donor of all compounds is zero, while the number of hydrogen bond acceptor ranges from 0 to 4. As for the MLogP value, all compounds have a value that is below 4.15, except for compound 3s. At this point, compounds 3o and 3p meet with the Lipinski’s rule of five, which have the potential to be developed as lead compounds for the therapy of cholinesterase inhibition related diseases.

**TABLE 2:** The predicted druggability of compounds **3o**, **3p**, and **3s** *^a^* ^*,*^ *^b^*.

Lipinski’s rule of five	3o	3p	3s
MW (<500 Da)	249.31 (249.31)	253.73 (253.73)	295.38 (295.38)
MLog *p* (<4.15)	3.11 (3.93)	4.04 (4.55)	4.69 (5.71)
HBD (5)	0 (0)	0 (0)	0 (0)
HBA (<10)	1 (2)	0 (1)	0 (1)
Violation	0	0	1

^a^ Values outside and within the parentheses were predicted by SwissADME, and the Molinspiration calculation software.

^b^ MW*:* molecular weight; HBD: hydrogen bond donor; HBA: hydrogen bond acceptor.

#### 2.2.3 Inhibition kinetics of butyrylcholinesterase

**FIGURE 2:**
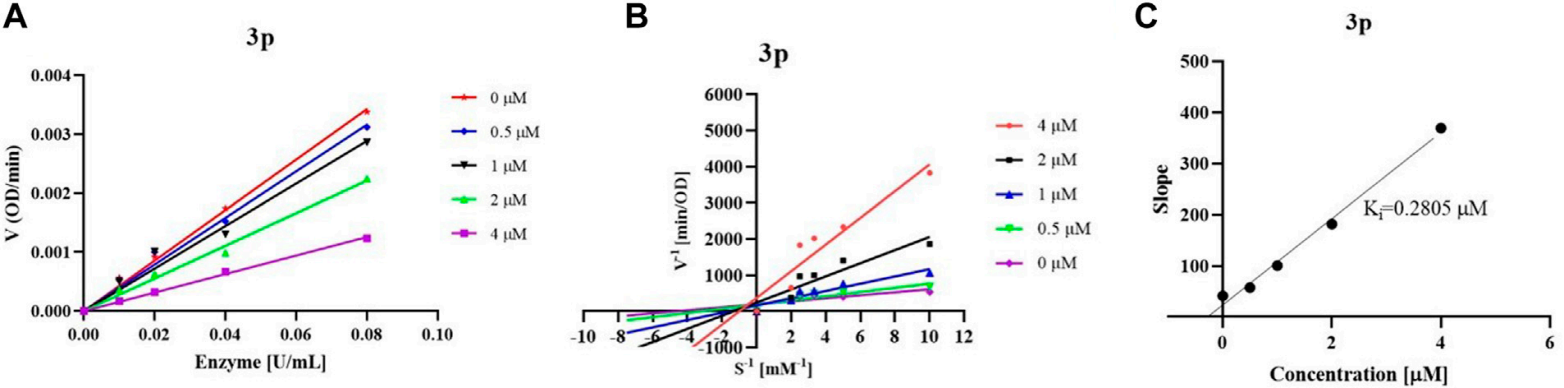
Inhibition type of compound **3p (A)**; Lineweaver–Burk plots of compound **3p (B)**; K_i_ values of compound **3p (C)**.

According to the type of interaction between inhibitor and enzyme, there are three reaction types including the reversible, irreversible, and pseudo-irreversible reactions in the cholinesterase catalyzed reaction, and each type has its representative characteristics. By studying the reaction rate changes in enzymatic reactions in the presence of inhibitors, the mechanism of action of inhibitors on enzymes, viz the inhibition type could be determined, which is of great significance for further structural modification and the discovery of compounds with better enzyme inhibitory activity. To reveal the interaction between synthesized inhibitors and *eq* BChE, we initially chose compound 3p as an example and studied the reversibility of enzymatic reaction in the presence of inhibitor. When the final concentration of substrate BTCI was fixed at 0.2 mM, we deduced from Figure 2A that both compound 3p increased the enzymatic reaction rate to varying degrees with the increase of enzyme concentration. When both the concentrations of *eq* BChE and BTCI remained constant, the rate of the enzymatic reaction decreased with the increase of inhibitor concentration. Overall, regardless of whether there was an inhibitor in this reaction system or not, a straight line through the origin was obtained, indicating that the inhibition of these compounds on *eq* BChE belongs to a reversible reaction, that was, their enzyme inhibition properties were accomplished through non-covalent binding with the enzyme.

In the process of reversible inhibition reaction, inhibitors can be divided into competitive, non-competitive, mixed competitive, and anti-competitive inhibitors according to the effect of inhibitors and substrate on enzyme (Liu et al., 2022). After having proved the reversible inhibition of these synthesized compounds, we then studied the mechanism of reversible inhibition of compound 3p on *eq* BChE by establishing the Lineweaver-Burk double reciprocal function. For compound 3p, the K_m_ values increased while the V_max_ values decreased compared with those in the absence of inhibitors, which was not in accordance with the characteristics of competitive, non-competitive, and anti-competitive inhibitors. Therefore, non-competitive in combination with competitive inhibition and/or non-competitive in combination with anti-competitive inhibition maybe existed in the reaction systems of compound 3p with enzyme (Figure 2B). (Hv et al., 2020) The above results indicated that compound 3p may combine with the free enzyme through the catalytic active sites (CAS) of enzyme to act as competitive inhibitors, or combine with enzyme together with substrate to act as non-competitive inhibiters, or directly interacte with the substrate-enzyme complex to act as anti-competitive inhibitors. However, the binding affinity of compounds 3p with the free enzyme or the substrate-enzyme complex was different. Subsequently, the inhibition constants (K_i_) of compound 3p on *eq*BChE were obtained by replotting slopes obtained from the Lineweaver-Burk double reciprocal function vs*.* concentration (Figure 2C), and the K_i_ values were consistent with the IC_50_ values.

#### 2.2.4 Molecular docking

**FIGURE 3:**
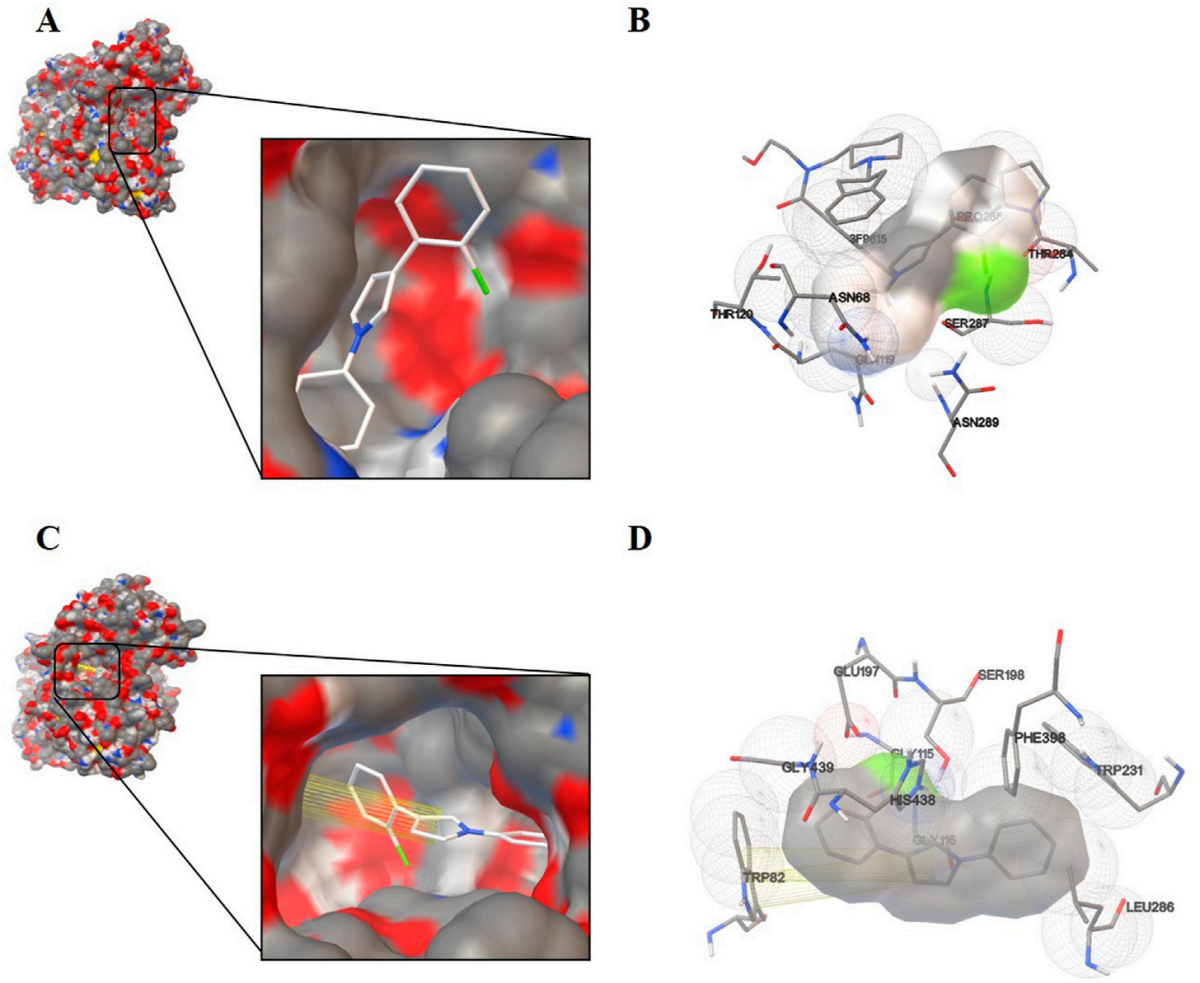
Schematic representation for the proposed binding mode of compound **3p** with BChE-3F9615 complex (PDB code: 4TPK) **(A)** or with BChE **(C)**. Compound **3p** was docked in the active site of BChE in the presence **(B)** or absence **(D)** of 3F9615. The co-crystallographic ligand is presented as 3F9615.

After having proved the inhibition mode of compound 3p with BChE, we performed molecular docking of compound 3p within the active site of BChE or BChE-3F9615 complex to further elucidate the binding mechanism (Brus et al., 2014; Jing et al., 2019). As shown in Figure 3, compound 3p was deeply inserted within the active-site gorge in cases with or without 3F9615, with the low binding energy of -6.64 and -6.93 kcal/mol (Table 3), respectively. It could interacte with the catalytic sites (SER198 and HIS438), acyl binding pocket (TRP231 and LEU286), and the choline binding pocket (TRP82) of BChE in the absence of 3F9615. Among them, the binding of this molecule with TRP82 was accomplished by pi-pi stacking interactions of the pyrrole ring with the two aryl rings of the indole section in TRP82 (Figures 3C,D). However, these interactions disappeared in the presence of 3F9615, but interactions with residues SER287 (close to Leu 286 and VAL288 in the acyl binding pocket) and ASN68 (close to ASP70 in the peripheral site) may affect the active site of BChE (Figures 3A,B). The above docking results well supported the mix inhibition of compound 3p.

**TABLE 3:** Molecular docking analysis of compound **3p** against the active sites of BChE*^a^*.

Compound	Binding energy (kcal/mol)	Ligand efficiency	Inhibition constant (μM)	Intermolecular energy	VDW-H*^b^* bond desolvation energy
**3p**	−6.64 (−6.93)	−0.37 (−0.39)	13.64 (8.33)	−7.23 (−7.53)	−7.25 (−7.5)

^a^ The data obtained with or without 3F9615 were presented outside and within parentheses.

^b^ VDW-H: Van der Walls-H-bond.

## 3 Conclusion

Enlightened by the fact that (hetero)aryl rings are prevalent in cholinesterase inhibitors and the pyrrole ring plays an important role in new drug discovery, the syntheses, biological activity, and mechanism of action of a series of pyrrole derivatives were described in this work. The results showed that compounds 3o, 3p, and 3s had good and selective inhibition on BChE with IC_50_ values ranging from 1.71 to 5.37 µM, which were comparable to the positive drug. Regarding to the druggability, the above three compounds met well with the Lipinsky’s five rules, except that compound 3s possesses a MLogP value of over 4.15. The inhibition kinetic studies revealed that compound 3p exhibited mixed competitive inhibition on BChE, which was consistent with the docking results that compound 3p interacted with the catalytic residues, the acyl binding pocket, and the choline binding pocket of BChE, or interacted with other residues that were close to the active site to exert mix-type bind mode with BChE. Collectively, the 1,3-diaryl-pyrrole scaffold could be used as a good BChE-targeted lead structure for future drug discovery.

## 4 Experiments

### 4.1 Chemistry

The characterization data of all compounds have been published in our previous work, and they were directly used after the purity of compounds were confirmed to be >95% by HPLC (Agilent 1260 Infinity II, United States). Mobile phase: 0–7 min MeOH:H_2_O = 20:80; 7–16 min MeOH:H_2_O = 95:5; 16–25 min MeOH:H_2_O = 20:80. Wavelength: 254 nm. The column was Eclipse Plus C18 (4.6 × 150 mm, 4 µm). Flow rate: 0.5 mL/min. Column temperature: 25°C. The HPLC spectra of compounds have been attached in Supporting Information S1.

### 4.2 Procedure for estimating the inhibitory activity against AChE and BChE by Ellman’s method

AChE (E.C. 3.1.1.7, Type V-S, lyophilized powder, from electric eel, 1000 unit), BChE (E.C. 3.1.1.8, from equine serum), ATCI, BTCI, and DTNB were purchased from Sigma-Aldrich. To the experimental group in a 96-well plate, 10 μl AChE or BChE (final concentration is 0.04 U/ml), 25 μl tested compounds, and 65 μl PBS buffer was sequentially added. For the control group, all components were in accordance with those in the experimental group, except that 25 μl tested compounds were replaced by equal volume of PBS. For the blank group, only 100 μl PBS was added. After incubating the above mixture in 37°C for a specific time, 100 μL DTNB (final concentration is 0.14 mM) and 50 μl ATCI or BTCI (final concentration 0.2 mM) were added in sequence. After reacting at room temperature for 6 min, the OD values were detected at a wavelength of 405 nm by using a microplate reader (TECAN spark, Austria). The calculation formula is: inhibition rate% = (OD_control_-OD_test_)/(OD_control_-OD_blank_)*100.

### 4.3 Details for prediction of the druggability of compounds

SwissADME (http://www.swissadme.ch/) and the Molinspiration calculation software (https://www.molinspiration.com/cgi-bin/properties) was used.

### 4.4 Details for molecular docking

The crystal structures of human butyrylcholinesterase in complex with N-((1-(2,3-dihydro-1H-inden-2-yl)piperidin-3-yl)methyl)-N-(2-methoxyethyl)-2-naphthamide (PDB ID: 4tpk, R = 2.70 Å) was downloaded from the PDB database (http://www.rcsb.org/) in. pdb format. The 3D structure for compound **3p** was prepared from Chem3D Pro 14.0. AutoDock 4.2.6 was employed to treat ligand and protein, perform autogrid and autodock, and analyze the docking results. Among them, autogrid was utilized to prepare the grid maps using a grid box size of 66 × 62 × 58 xyz points with a spacing of 0.375 angstrom, and the center of the grid box (x = 3.157, y = 10.171, and z = 16.804). The parameter of grid box is determined in the premise of including all the residues in the active-site gorge of BChE (Brus et al., 2014; Jing et al., 2019). For the docking step, the Lamarckian genetic algorithm and the pseudo-Solis and Wets methods were applied for minimization, using default parameters; semi-flexible docking (the conformations of ligand and protein are flexible and rigid, respectively) was used; the number of GA runs was 50, and other parameters were all directly used.

## Data Availability

The original contributions presented in the study are included in the article/Supplementary Material, further inquiries can be directed to the corresponding authors.
