# Supplementary material for: Discovery of pyrrole derivatives as acetylcholinesterase-sparing butyrylcholinesterase inhibitor
Source: Front Pharmacol. 2022 Dec 6;13:1043397. doi: 10.3389/fphar.2022.1043397 (PMC9763612; doi:10.3389/fphar.2022.1043397)

Supporting Information

Discovery of Pyrrole Derivatives as Acetylcholinesterase-Sparing Butyrylcholinesterase Inhibitor

Shouyuan Sun,[a,#] Tao Shi,[b,#] Yan Peng,[c] Honghua Zhang,[b] Linsheng Zhuo,[c] Xue Peng,[c] Qien Li,[d,e] Manxia Wang,[a*] Shuzhi Wang,[c,*] and Zhen Wang[c,b,*]

[a]*Lanzhou University Second Hospital, Lanzhou 730030, China*

[b]*School of Pharmacy, Lanzhou University, No 199 West Donggang Road, Lanzhou 730000, China.*

[c]*School of Pharmaceutical Science, Hengyang Medical School, University of South China, No 28 West Changsheng Road, Hengyang 421001, China.*

[d]*Tibetan Medical College, Qinghai University, Xining 810016, Qinghai, China*

[e] *State Key Laboratory of Tibetan Medicine Research and Development, Xining 810016, Qinghai, China*

*E-mail for corresponding authors: wmx322@aliyun.com; zhenw@lzu.edu.cn; shuzhi.wang@usc.edu.cn

[#]S. Sun and T. Shi contributed equally to this work.

Table of Contents

1. NMR data of compounds **2**

12. Purity of compounds **5**

3. Screen of the most appropriate incubation time for compounds and enzymes **13**

4. SMILE for compounds **15**

5. NMR spectra and data for synthesized compounds **16**

1. NMR data of compounds

1-(o-Tolyl)-1H-pyrrole (3a): ^{1}H NMR (400 MHz, $CDCl_3$) δ 7.29 – 7.21 (m, 4H), 6.80 – 6.73 (m, 2H), 6.34 – 6.27 (m, 2H), 2.20 (s, 3H). ^{13}C NMR (101 MHz, $CDCl_3$) δ 140.52, 133.72, 130.93, 127.37, 126.52, 126.42, 121.93, 108.62, 17.74.

1-(2-Chlorophenyl)-1H-pyrrole (3b): ^{1}H NMR (400 MHz, $CDCl_3$) δ 7.53 – 7.49 (m, 1H), 7.37 – 7.32 (m, 2H), 7.31 – 7.26 (m, 1H), 6.92 (t, *J* = 2.1 Hz, 2H), 6.35 (t, *J* = 2.1 Hz, 2H). ^{13}C NMR (101 MHz, $CDCl_3$) δ 138.69, 130.62, 129.56, 128.13, 127.75, 127.47, 122.10, 109.21.

1-(m-Tolyl)-1H-pyrrole (3c): ^{1}H NMR (400 MHz, $CDCl_3$) δ 7.31 (t, *J* = 7.6 Hz, 1H), 7.23 – 7.18 (m, 2H), 7.10 – 7.04 (m, 3H), 6.34 (t, *J* = 2.1 Hz, 2H), 2.41 (s, 3H). ^{13}C NMR (75 MHz, $CDCl_3$) δ 140.68, 139.46, 129.26, 126.34, 121.25, 119.29, 117.62, 110.13, 21.43.

1-(3-Bromophenyl)-1H-pyrrole (3d): ^{1}H NMR (400 MHz, $CDCl_3$) δ 7.54 (t, *J* = 1.9 Hz, 1H), 7.37 – 7.23 (m, 3H), 7.05 (t, *J* = 2.2 Hz, 2H), 6.34 (t, *J* = 2.2 Hz, 2H). ^{13}C NMR (101 MHz, $CDCl_3$) δ 141.79, 130.73, 128.42, 123.43, 123.03, 119.11, 118.81, 110.95.

1-(4-Isopropylphenyl)- pyrrole (3e): ^{1}H NMR (400 MHz, $CDCl_3$) δ 7.34 – 7.30 (m, 2H), 7.29 – 7.26 (m, 2H), 7.06 (t, *J* = 2.2 Hz, 2H), 6.33 (t, *J* = 2.2 Hz, 2H), 2.94 (hept, *J* = 6.9 Hz, 1H), 1.27 (d, *J* = 6.9 Hz, 6H). ^{13}C NMR (101 MHz, $CDCl_3$) δ 146.33, 138.60, 127.35, 120.57, 119.35, 109.92, 33.53, 23.97.

1-(4-(Methylthio)phenyl)-1H-pyrrole(3f): ^{1}H NMR (400 MHz, $CDCl_3$) δ 7.32 (s, 4H), 7.06 (t, *J* = 2.2 Hz, 2H), 6.34 (t, *J* = 2.2 Hz, 2H), 2.51 (s, 3H). ^{13}C NMR (101 MHz, $CDCl_3$) δ 138.23, 135.38, 127.99, 120.97, 119.20, 110.32, 16.36.

1-(4-Bromophenyl)-1H-pyrrole (3g): ^{1}H NMR (400 MHz, $CDCl_3$) δ 7.56 – 7.51 (m, 2H), 7.29 – 7.25 (m, 2H), 7.05 (t, *J* = 2.2 Hz, 2H), 6.36 (t, *J* = 2.2 Hz, 2H). ^{13}C NMR (101 MHz, $CDCl_3$) δ 139.65, 132.49, 121.84, 119.11, 118.61, 110.79.

Methyl 4-(1H-pyrrol-1-yl)benzoate (3h): ^{1}H NMR (400 MHz, $CDCl_3$) δ 8.13 – 8.04 (m, 2H), 7.46 – 7.40 (m, 2H), 7.18 – 7.12 (m, 2H), 6.43 – 6.33 (m, 2H), 3.92 (s, 3H). ^{13}C NMR (101 MHz, $CDCl_3$) δ 166.29, 143.86, 131.19, 126.79, 119.16, 118.90, 111.37, 52.05.

1-(2,6-Dimethylphenyl)-pyrrole (3i): ^{1}H NMR (400 MHz, $CDCl_3$) δ 7.22 – 7.16 (m, 1H), 7.11 (d, *J* = 7.4 Hz, 2H), 6.60 (t, *J* = 2.1 Hz, 2H), 6.32 (t, *J* = 2.1 Hz, 2H), 2.03 (s, 6H). ^{13}C NMR (101 MHz, $CDCl_3$) δ 139.83, 136.15, 127.85, 127.83, 121.23, 108.41, 17.22.

2-(1H-Pyrrol-1-yl)pyridine (3j): ^{1}H NMR (400 MHz, $CDCl_3$) δ 8.45 – 8.41 (m, 1H), 7.77 – 7.71 (m, 1H), 7.53 – 7.51 (m, 2H), 7.32 (dt, *J* = 8.4, 0.9 Hz, 1H), 7.13 – 7.08 (m, 1H), 6.38 – 6.34 (m, 2H). ^{13}C NMR (101 MHz, $CDCl_3$) δ 151.29, 148.62, 138.42, 120.08, 117.98, 111.34, 111.23.

5-Methyl-3-(1H-pyrrol-1-yl)isoxazole (3k): ^{1}H NMR (400 MHz, $CDCl_3$) δ 7.17 (t, *J* = 2.2 Hz, 2H), 6.34 (t, *J* = 2.2 Hz, 2H), 6.11 (s, 1H), 2.46 (s, 3H). ^{13}C NMR (101 MHz, $CDCl_3$) δ 171.02, 159.36, 118.90, 111.42, 93.99, 12.69. HRMS (ESI, m/z) $[M + Na]^{+}$ calcd for $C_8H_8N_2NaO^+$ 171.0529, found 171.0531.

2-(1H-Pyrrol-1-yl)quinoxaline (3l): ^{1}H NMR (400 MHz, $CDCl_3$) δ 9.12 (s, 1H), 8.06 (d, *J* = 8.3 Hz, 1H), 7.99 (d, *J* = 8.3 Hz, 1H), 7.79 – 7.74 (m, 1H), 7.74 – 7.71 (m, 2H), 7.70 – 7.65 (m, 1H), 6.47 (t, *J* = 2.3 Hz, 2H). ^{13}C NMR (101 MHz, $CDCl_3$) δ 144.97, 140.85, 140.42, 136.47, 130.92, 129.10, 128.33, 128.27, 118.24, 112.66. HRMS (ESI, m/z) $[M + Na]^{+}$ calcd for $C_{12}H_9N_3Na^+$ 218.0689, found 218.0691.

3-Benzyl-1-phenyl-1H-pyrrole (3m): ^{1}H NMR (400 MHz, $CDCl_3$) δ 7.40 – 7.36 (m, 2H), 7.35 – 7.32 (m, 2H), 7.30 – 7.27 (m, 4H), 7.22 – 7.17 (m, 2H), 7.02 (t, *J* = 2.5 Hz, 1H), 6.84 (s, 1H), 6.21 – 6.15 (m, 1H), 3.88 (s, 2H). ^{13}C NMR (101 MHz, $CDCl_3$) δ 141.74, 140.62, 129.39, 128.62, 128.27, 125.76, 125.46, 125.15, 119.95, 119.17, 117.16, 111.14, 33.48.

1,3-Diphenyl-1H-pyrrole (3n): ^{1}H NMR (400 MHz, $CDCl_3$) δ 7.60 – 7.56 (m, 2H), 7.45 – 7.42 (m, 4H), 7.40 – 7.34 (m, 3H), 7.30 – 7.25 (m, 1H), 7.23 – 7.18 (m, 1H), 7.12 (dd, *J* = 3.0, 2.2 Hz, 1H), 6.66 (dd, *J* = 2.9, 1.8 Hz, 1H). ^{13}C NMR (101 MHz, $CDCl_3$) δ 140.41, 135.20, 129.54, 128.61, 126.75, 125.76, 125.71, 125.10, 120.26, 120.24, 115.71, 108.62.

3-(2-Methoxyphenyl)-1-phenyl-1H-pyrrole (3o): ^{1}H NMR (400 MHz, $CDCl_3$) δ 7.64 (t, *J* = 2.0 Hz, 1H), 7.58 (dd, *J* = 7.6, 1.8 Hz, 1H), 7.46 – 7.39 (m, 4H), 7.26 – 7.21 (m, 1H), 7.19 (ddd, *J* = 9.0, 7.4, 1.7 Hz, 1H), 7.11 (t, *J* = 2.6 Hz, 1H), 7.01 – 6.94 (m, 2H), 6.75 (dd, *J* = 3.0, 1.7 Hz, 1H), 3.91 (s, 3H). ^{13}C NMR (101 MHz, $CDCl_3$) δ 156.05,

140.66, 129.43, 127.79, 126.56, 125.45, 123.98, 122.27, 120.71, 120.31, 119.06, 119.04, 111.05, 110.17, 55.31. HRMS (ESI, m/z) [M + Na] + calcd for $C_{17}H_{15}NNaO^+$ 272.1046, found 272.1033.

3-(2-Chlorophenyl)-1-phenyl-1H-pyrrole (3p): ^{1}H NMR (400 MHz, $CDCl_3$) δ 7.55 – 7.52 (m, 2H), 7.46 – 7.42 (m, 5H), 7.29 – 7.24 (m, 2H), 7.18 – 7.12 (m, 2H), 6.69 (dd, *J* = 3.0, 1.8 Hz, 1H). ^{13}C NMR (101 MHz, $CDCl_3$) δ 140.35, 134.03, 131.66, 130.32, 130.04, 129.54, 126.89, 126.76, 125.82, 123.52, 120.41, 119.19, 119.04, 111.30. HRMS (ESI, m/z) [M + Na] + calcd for $C_{16}H_{12}ClNNa^+$ 276.0550, found 276.0557.

3-(3-Methoxyphenyl)-1-phenyl-1H-pyrrole (3q): ^{1}H NMR (400 MHz, $CDCl_3$) δ 7.45 – 7.40 (m, 4H), 7.37 (t, *J* = 2.0 Hz, 1H), 7.30 – 7.22 (m, 2H), 7.17 (dt, *J* = 7.8, 1.2 Hz, 1H), 7.12 – 7.09 (m, 2H), 6.76 (ddd, *J* = 8.2, 2.6, 1.0 Hz, 1H), 6.64 (dd, *J* = 2.9, 1.8 Hz, 1H), 3.84 (s, 3H). ^{13}C NMR (101 MHz, $CDCl_3$) δ 159.88, 140.41, 136.70, 129.57, 129.53, 126.67, 125.73, 120.27, 120.22, 117.78, 115.91, 111.11, 110.85, 108.74, 55.13. HRMS (ESI, m/z) [M + Na] + calcd for $C_{17}H_{15}NNaO^+$ 272.1046, found 272.1038.

1-Phenyl-3-(3-(trifluoromethyl)phenyl)-1H-pyrrole (3r): ^{1}H NMR (400 MHz, $CDCl_3$) δ 7.79 (s, 1H), 7.74 – 7.70 (m, 1H), 7.47 – 7.40 (m, 7H), 7.31 – 7.25 (m, 1H), 7.14 – 7.11 (m, 1H), 6.66 (dd, *J* = 3.0, 1.8 Hz, 1H). ^{13}C NMR (101 MHz, CDCl3) δ 140.21, 136.08, 131.07, 130.75, 129.61, 129.01, 128.21 – 128.07 (m), 126.05, 125.42, 122.23 (q, *J* = 3.8 Hz), 121.66 (q, *J* = 3.8 Hz), 120.71, 120.40, 116.29, 108.53. HRMS (ESI, m/z) [M + Na] + calcd for $C_{17}H_{12}F_3NNa^+$ 310.0814, found 310.0812.

3-([1,1'-Biphenyl]-4-yl)-1-phenyl-1H-pyrrole (3s): ^{1}H NMR (400 MHz, $CDCl_3$) δ 7.67 – 7.60 (m, 6H), 7.48 – 7.42 (m, 7H), 7.36 – 7.31 (m, 1H), 7.31 – 7.27 (m, 1H), 7.15 (t, *J* = 2.6 Hz, 1H), 6.70 (dd, *J* = 3.0, 1.8 Hz, 1H). ^{13}C NMR (101 MHz, $CDCl_3$) δ 140.91, 140.42, 138.46, 134.31, 129.57, 128.68, 127.32, 126.94, 126.78, 126.35, 125.77, 125.44, 120.37, 120.29, 115.81, 108.67. HRMS (ESI, m/z) [M + Na] + calcd for $C_{22}H_{17}NNa^+$ 318.1253, found 318.1250.

2. Purity of compounds

Summary of purity of synthesized compounds

Compounds	Purity
3a	95.4063%
3b	97.4963%
3c	98.1426%
3d	99.6624%
3e	98.9497%
3f	96.9960%
3g	98.4890%
3h	100.0000%
3i	96.8207%
3j	100.0000%
3k	96.8170%
3l	96.7221%
3m	95.5761%
3n	99.3678%
3o	98.2417%
3p	97.5287%
3q	97.7737%
3r	97.8092%

compound 3a (95.4063%)

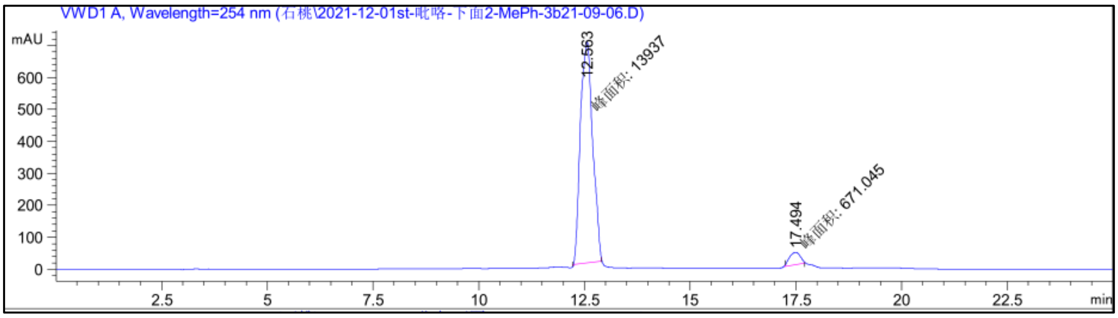

Peak	RetTime [min]	Type	Width [min]	Area [mAU*s]	Height [mAU]	Area %
1	12.563	MM	0.3357	1.39370e4	691.83929	95.4063
2	17.494	MM	0.2982	671.04486	37.50217	4.5937

compound 3b (97.4963%)

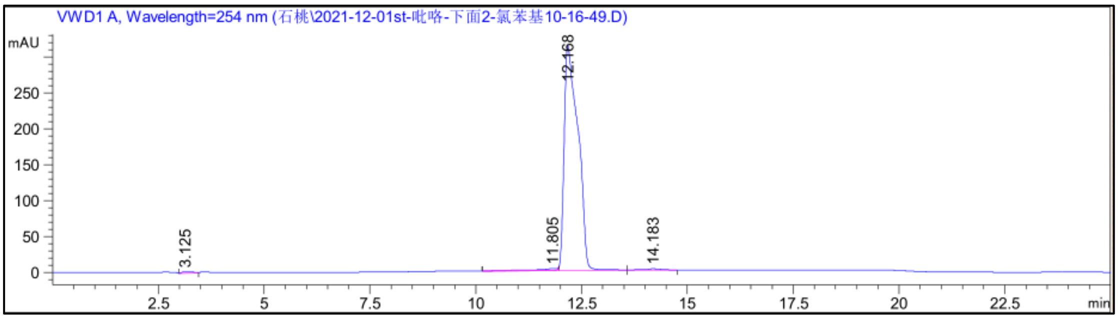

Peak	RetTime [min]	Type	Width [min]	Area [mAU*s]	Height [mAU]	Area %
1	3.125	BV	0.2663	34.41362	1.80585	0.4732
2	11.805	BV E	0.4778	98.78674	2.65676	1.3584
3	12.168	VV R	0.3064	7090.10010	312.87875	97.4963
4	14.183	BB	0.3850	48.87122	1.83337	0.6720

compound 3c (98.1426%)

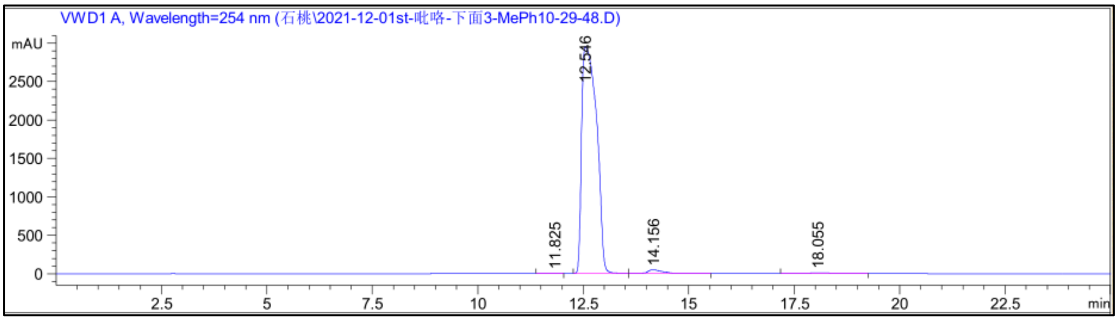

Peak	RetTime [min]	Type	Width [min]	Area [mAU*s]	Height [mAU]	Area %
1	11.825	BV	0.2632	33.88391	1.94569	0.0461
2	12.546	BB	0.4236	7.21921e4	2949.47583	98.1426
3	14.156	BV R	0.3689	1090.57239	44.97609	1.4826
4	18.055	VB R	0.4916	241.84819	6.78910	0.3288

compound 3d (99.6624%)

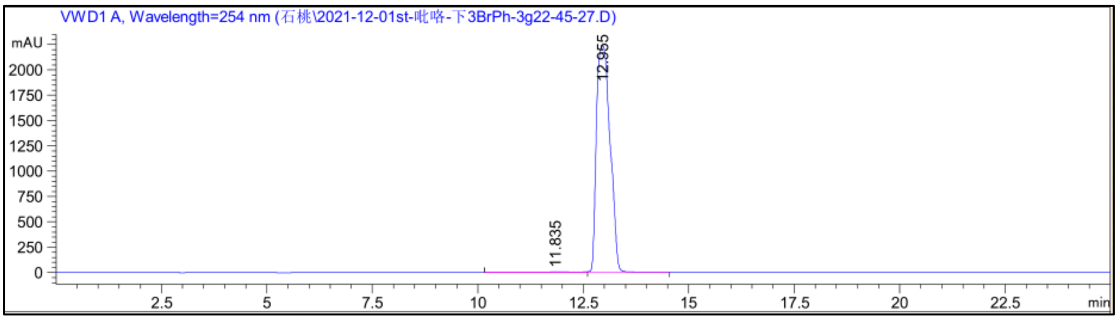

Peak	RetTime [min]	Type	Width [min]	Area [mAU*s]	Height [mAU]	Area %
1	11.835	BV E	0.5579	170.05333	3.83113	0.3376
2	12.955	VV R	0.3496	5.02031e4	2238.52539	99.6624

compound 3e (98.9497%)

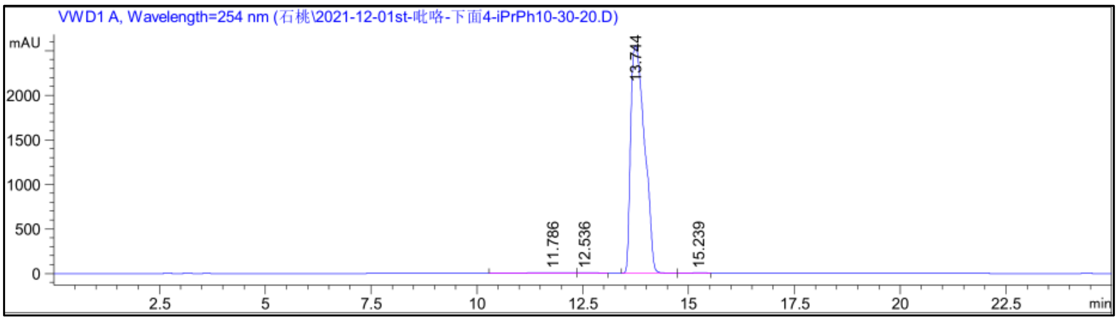

Peak	RetTime [min]	Type	Width [min]	Area [mAU*s]	Height [mAU]	Area %
1	11.786	BV	0.7832	394.72122	6.47491	0.6743
2	12.536	VB	0.4292	146.62666	5.83487	0.2505
3	13.744	BB	0.3547	5.79216e4	2552.76465	98.9497
4	15.239	BV	0.3636	73.44045	3.13130	0.1255

compound 3f (96.9960%)

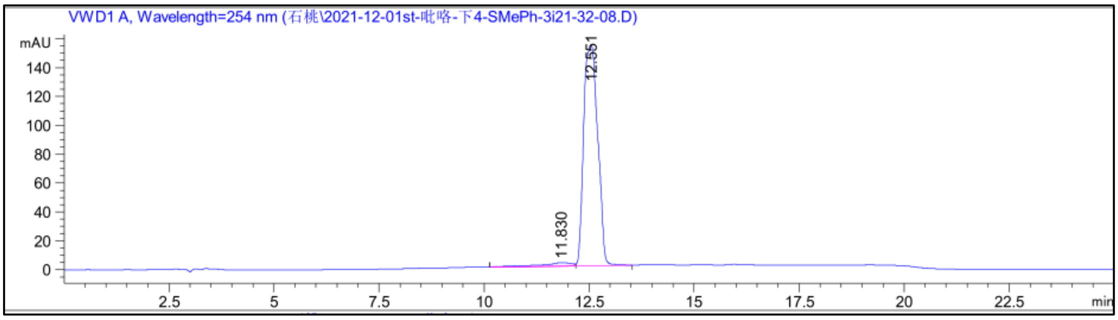

Peak	RetTime [min]	Type	Width [min]	Area [mAU*s]	Height [mAU]	Area %
1	11.830	BV E	0.6717	110.18739	2.26424	3.0040
2	12.551	VB R	0.3583	3557.85498	152.44003	96.9960

compound 3g (98.4890%)

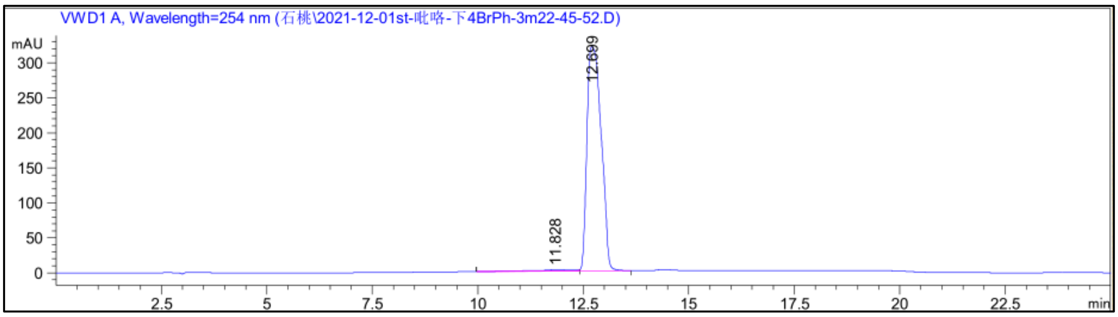

Peak	RetTime [min]	Type	Width [min]	Area [mAU*s]	Height [mAU]	Area %
1	11.828	BV E	0.7791	115.39997	2.01811	1.5110
2	12.699	VB R	0.3621	7521.72607	320.16687	98.4890

compound 3h (100.0000%)

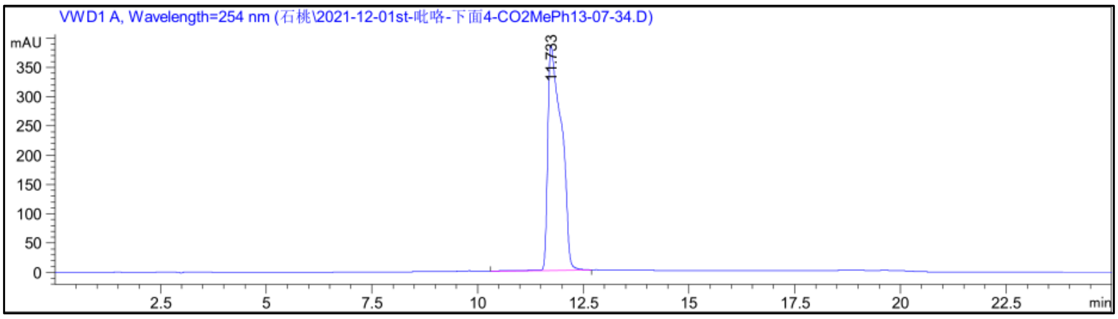

Peak	RetTime [min]	Type	Width [min]	Area [mAU*s]	Height [mAU]	Area %
1	11.733	BB	0.2980	8553.17871	383.81934	100.0000

compound 3i (96.8207%)

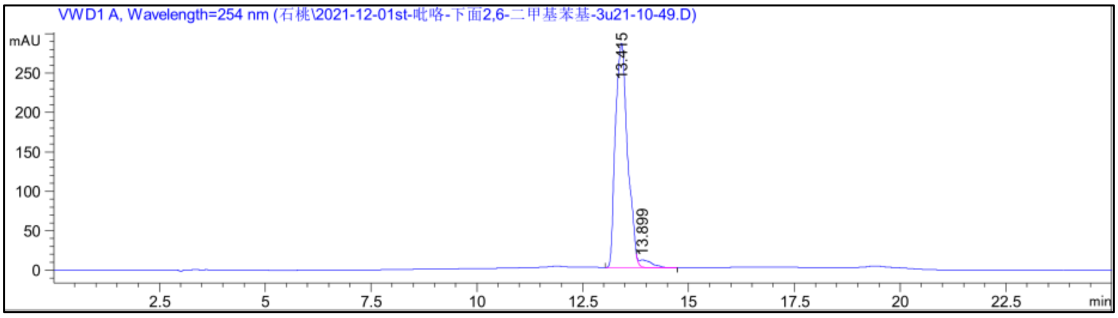

Peak	RetTime [min]	Type	Width [min]	Area [mAU*s]	Height [mAU]	Area %
1	13.415	BV R	0.3165	5712.57910	284.23669	96.8207
2	13.899	VB E	0.2737	187.58229	8.83660	3.1793

compound 3j (100.0000%)

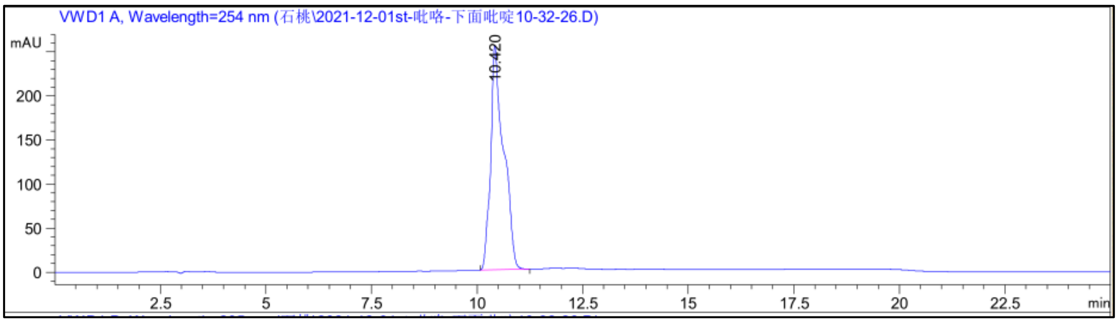

Peak	RetTime [min]	Type	Width [min]	Area [mAU*s]	Height [mAU]	Area %
1	10.420	BB	0.2831	5435.84326	254.62431	100.0000

compound 3k (96.8170%)

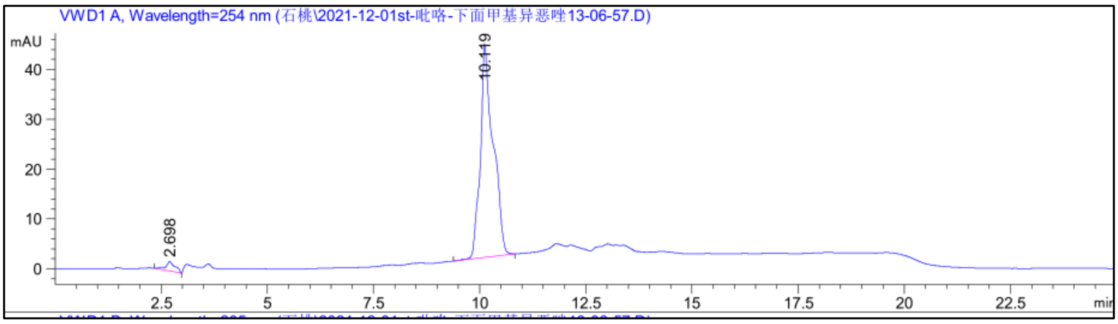

Peak	RetTime [min]	Type	Width [min]	Area [mAU*s]	Height [mAU]	Area %
1	2.698	BB	0.2097	29.39585	1.88797	3.1830
2	10.119	BB	0.2781	894.13269	42.74255	96.8170

compound 3l (96.7221%)

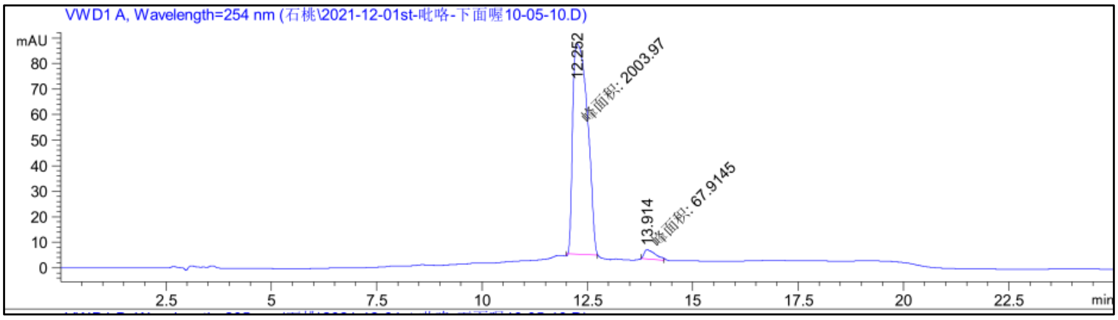

Peak	RetTime [min]	Type	Width [min]	Area [mAU*s]	Height [mAU]	Area %
1	12.252	MM	0.4049	2003.97473	82.48892	96.7221
2	13.914	MM	0.3112	67.91448	3.63730	3.2779

compound 3m (95.5761%)

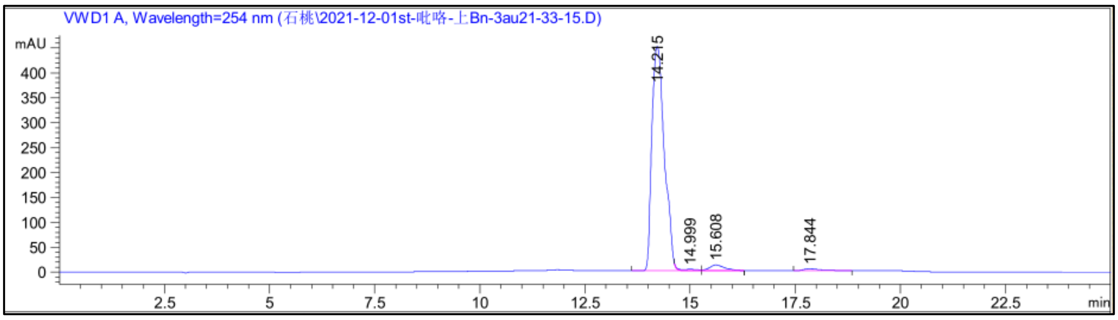

Peak	RetTime [min]	Type	Width [min]	Area [mAU*s]	Height [mAU]	Area %
1	14.215	BV R	0.3230	9290.57324	449.69522	95.5761
2	14.999	VV E	0.3965	66.70444	2.44346	0.6862
3	15.608	VB E	0.3687	268.46222	11.00116	2.7618
4	17.844	BB	0.4225	94.86727	3.43224	0.9759

compound 3n (99.3678%)

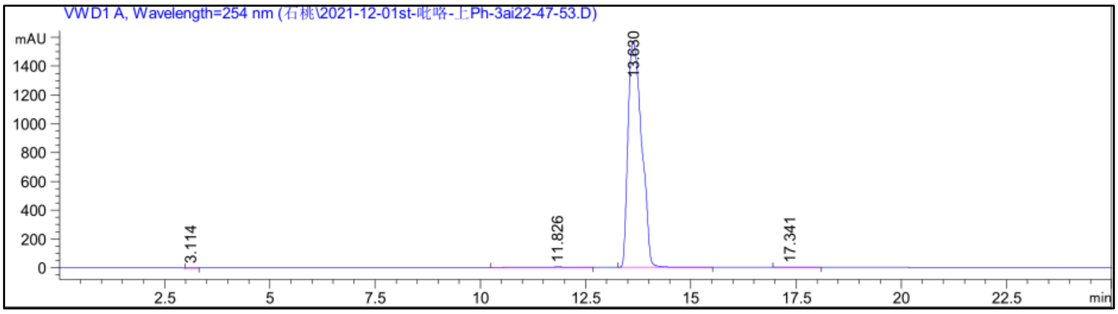

Peak	RetTime [min]	Type	Width [min]	Area [mAU*s]	Height [mAU]	Area %
1	3.114	BV	0.2114	25.30356	1.72255	0.0712
2	11.826	BB	0.7602	147.12108	2.71504	0.4142

3	13.630	BV R	0.3510	3.52963e4	1565.68701	99.3678
4	17.341	BB	0.3760	52.12784	2.06830	0.1468

compound 3o (98.2417%)

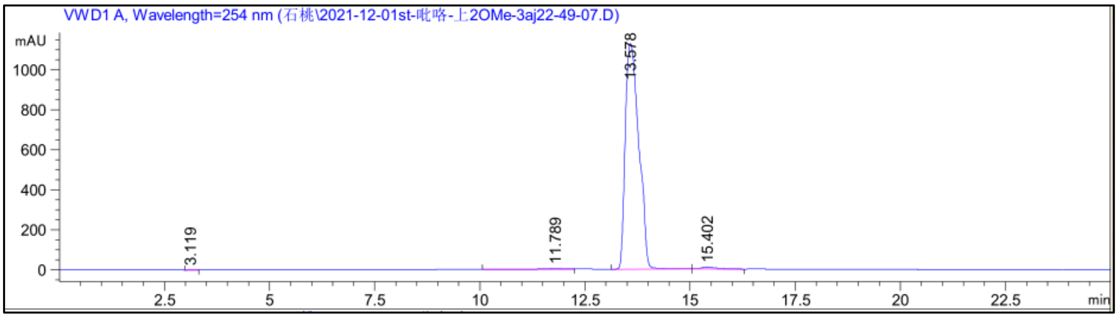

Peak	RetTime [min]	Type	Width [min]	Area [mAU*s]	Height [mAU]	Area %
1	3.119	BV	0.2077	25.42398	1.76921	0.0992
2	11.789	BV	0.5240	175.74423	4.30731	0.6860
3	13.578	BB	0.3505	2.51698e4	1127.04578	98.2417
4	15.402	BB	0.3895	249.32169	9.58417	0.9731

compound 3p (97.5287%)

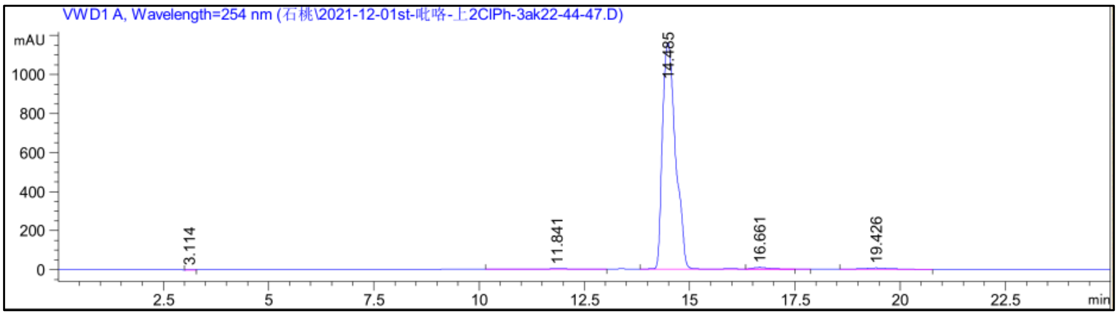

Peak	RetTime [min]	Type	Width [min]	Area [mAU*s]	Height [mAU]	Area %
1	3.114	BV	0.1904	23.31957	1.78512	0.0906
2	11.841	BB	0.9221	129.94298	1.87593	0.5050
3	14.485	BV R	0.3408	2.50960e4	1158.33057	97.5287
4	16.661	VB E	0.3910	202.10223	8.10388	0.7854
5	19.426	BB	0.6041	280.54886	6.70600	1.0903

compound 3q (97.7737%)

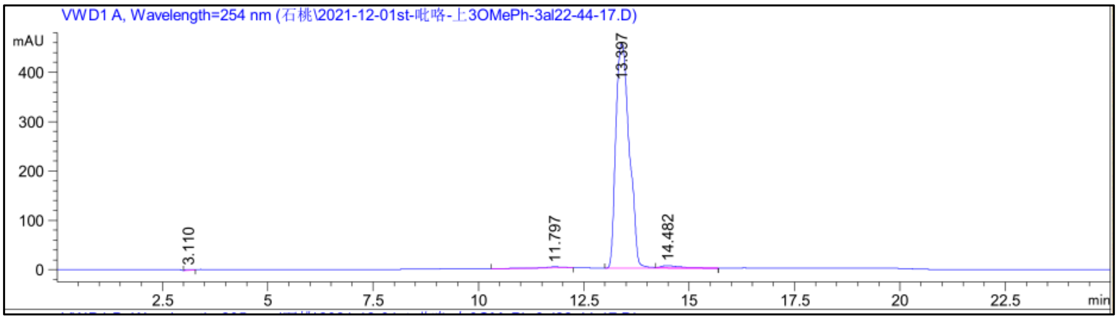

Peak	RetTime [min]	Type	Width [min]	Area [mAU*s]	Height [mAU]	Area %
1	3.110	BV	0.1841	23.41324	1.84415	0.2266
2	11.797	BB	0.3681	63.38784	2.26769	0.6135
3	13.397	BV R	0.3433	1.01018e4	454.56949	97.7737
4	14.482	VB E	0.4356	143.22166	4.83499	1.3862

compound 3r (97.8092%)

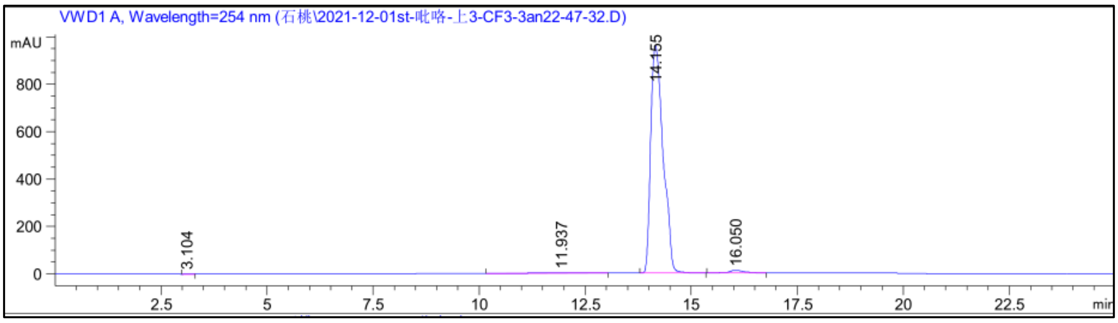

Peak	RetTime [min]	Type	Width [min]	Area [mAU*s]	Height [mAU]	Area %
1	3.104	BV	0.2042	24.92688	1.74940	0.1252
2	11.937	BB	0.8384	127.71622	2.05121	0.6417
3	14.155	BB	0.3149	1.94667e4	958.72101	97.8092
4	16.050	BB	0.3747	283.37811	11.21971	1.4238

3. Screen of the most appropriate incubation time for compounds and enzymes

In order to find the most appropriate incubation time for compounds and enzyme, to verify whether the incubation time of 25 min is appropriate, and to further improve the enzyme inhibition activity of these compounds, we then tested the inhibition rates of the above three optimal compounds at different concentrations in different incubation time, made the inhibition rate-incubation time curves (**Figure S1**), and calculated the IC_{50} values of these compounds under different incubation time (**Table S1**). As a result, with the extension of the incubation time, the enzyme activity gradually decreased and reached a steady state, and most compounds have reached the steady state when the incubation time was 25 min, which proved that the incubation time selected was appropriate. It could be seen from **Table S1** that when incubating compound **3o** with enzyme for 5 min, the IC_{50} value was the smallest, reaching 4.80 ± 0.38 μM. As for compound **3p**, the IC_{50} value was as small as 0.48 ± 0.24 μM at the incubation time of 10 min. When the incubation time of compound **3s** was 25 min, the IC_{50} value of *eq*BChE inhibitory activity reached 3.76 ± 0.25 μM.

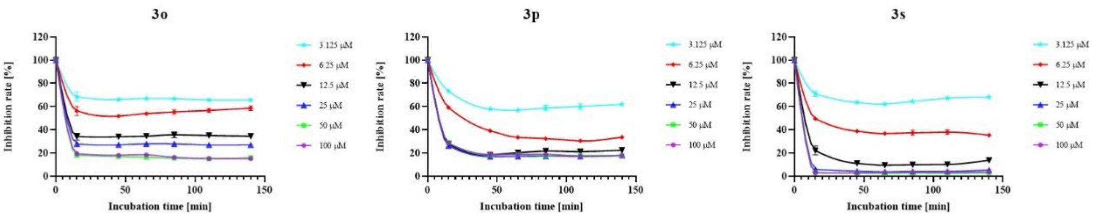

Figure S1. Inhibition rate-incubation time curves of compounds **3o**, **3p**, and **3s**.

Table S2. IC_{50} values of compounds under different incubation time.[a]

Incubation time	3o	3p	3s	Donepezil
**5 min**	4.80±0.38	0.60±0.10	5.87±0.23	1.25±0.04
**10 min**	5.06±1.20	0.48±0.24	5.95±0.30	1.32±0.097
**15 min**	6.87±0.60	1.95±0.13	6.05±0.65	1.24±0.12
**25 min**	5.37±0.36	1.71±0.087	3.76±0.25	1.58±0.066
**45 min**	6.67±0.61	2.40±0.11	4.41±0.15	1.32±0.070
**65 min**	6.83±0.24	3.27±0.49	4.21±0.075	1.29±0.041
**85 min**	7.63±0.77	3.20±0.092	4.38±0.18	1.33±0.018

**110 min**	8.96±0.82	4.94±0.55	4.63±0.22	1.39±0.063
**140 min**	7.92±0.51	7.62±0.43	5.09±0.16	1.30±0.12

[a]The final concentrations of BTCI (0.2 mM) and BChE (0.04 U/mL) were fixed. The results were expressed as mean±SD of at least three independent experiments.

4. SMILE for compounds

**Compound structure**	**SMILES**
**3a**	CC1=CC=CC=C1N1C=CC=C1
**3b**	ClC1=CC=CC=C1N1C=CC=C1
**3c**	CC1=CC(=CC=C1)N1C=CC=C1
**3d**	BrC1=CC(=CC=C1)N1C=CC=C1
**3e**	COC1=CC=C(C=C1)N1C=CC=C1
**3f**	CSC1=CC=C(C=C1)N1C=CC=C1
**3g**	BrC1=CC=C(C=C1)N1C=CC=C1
**3h**	COC(=O)C1=CC=C(C=C1)N1C=CC=C1
**3i**	CC1=CC=CC(C)=C1N1C=CC=C1
**3j**	C1=CN(C=C1)C1=CC=CC=N1
**3k**	CC1=CC(=NO1)N1C=CC=C1
**3l**	C1=CN(C=C1)C1=NC2=C(C=CC=C2)N=C1
**3m**	C(C1=CN(C=C1)C1=CC=CC=C1)C1=CC=CC=C1
**3n**	C1=CC(=CN1C1=CC=CC=C1)C1=CC=CC=C1
**3o**	COC1=C(C=CC=C1)C1=CN(C=C1)C1=CC=CC=C1
**3p**	ClC1=C(C=CC=C1)C1=CN(C=C1)C1=CC=CC=C1
**3q**	COC1=CC=CC(=C1)C1=CN(C=C1)C1=CC=CC=C1
**3r**	FC(F)(F)C1=CC=CC(=C1)C1=CN(C=C1)C1=CC=CC=C1
**3s**	C1=CC(=CN1C1=CC=CC=C1)C1=CC=C(C=C1)C1=CC=CC=C1

5. NMR spectra and data for synthesized compounds

¹H NMR spectrum of compound 3a (400 MHz, $CDCl_3$)

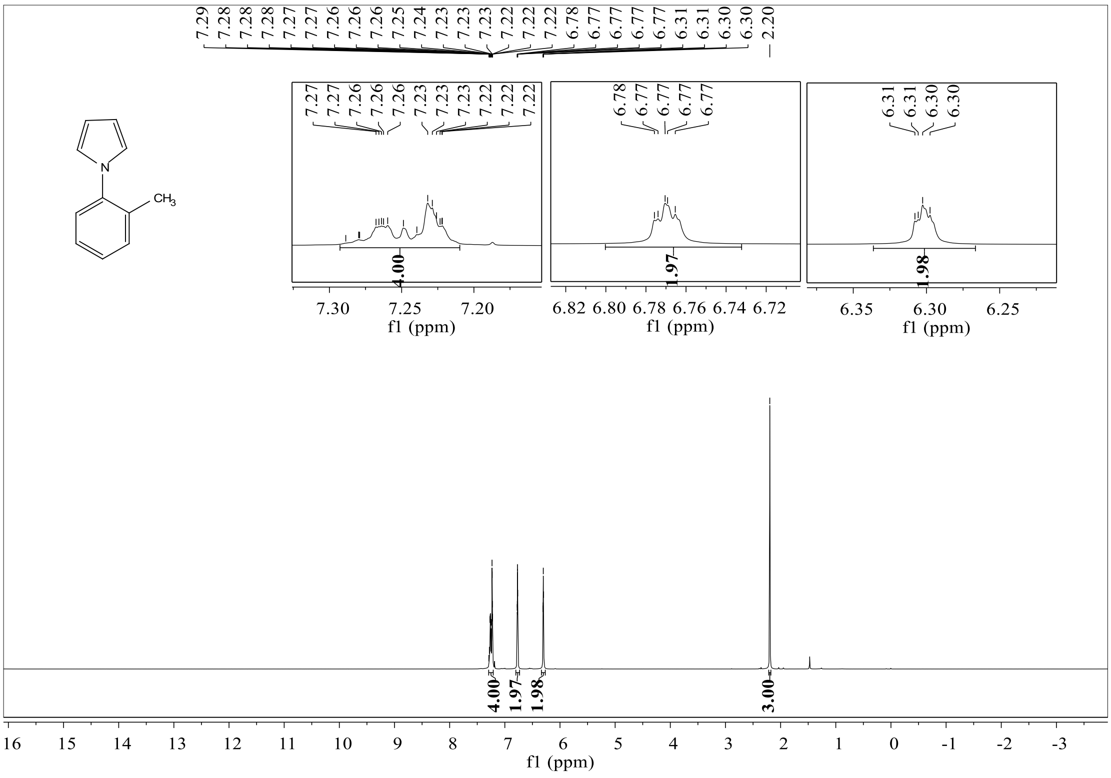

¹³C NMR spectrum of compound 3a (101 MHz, $CDCl_3$)

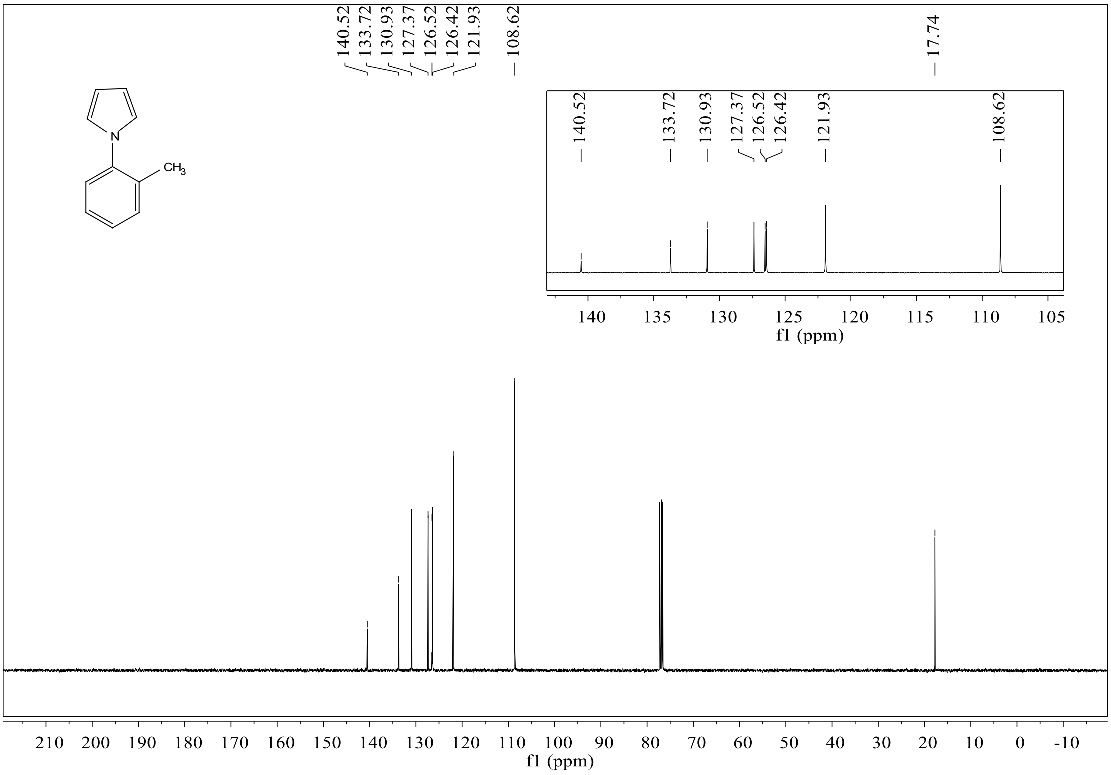

^{1}H NMR spectrum of compound 3b (400 MHz, $CDCl_3$)

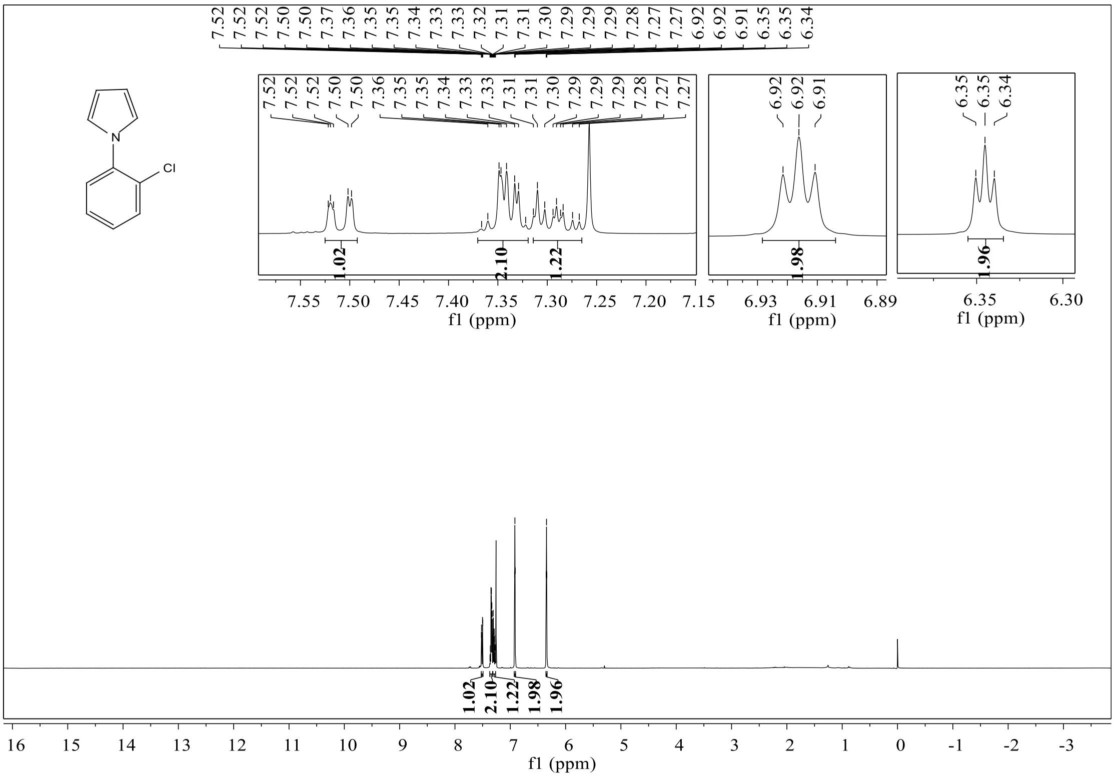

^{13}C NMR spectrum of compound 3b (101 MHz, $CDCl_3$)

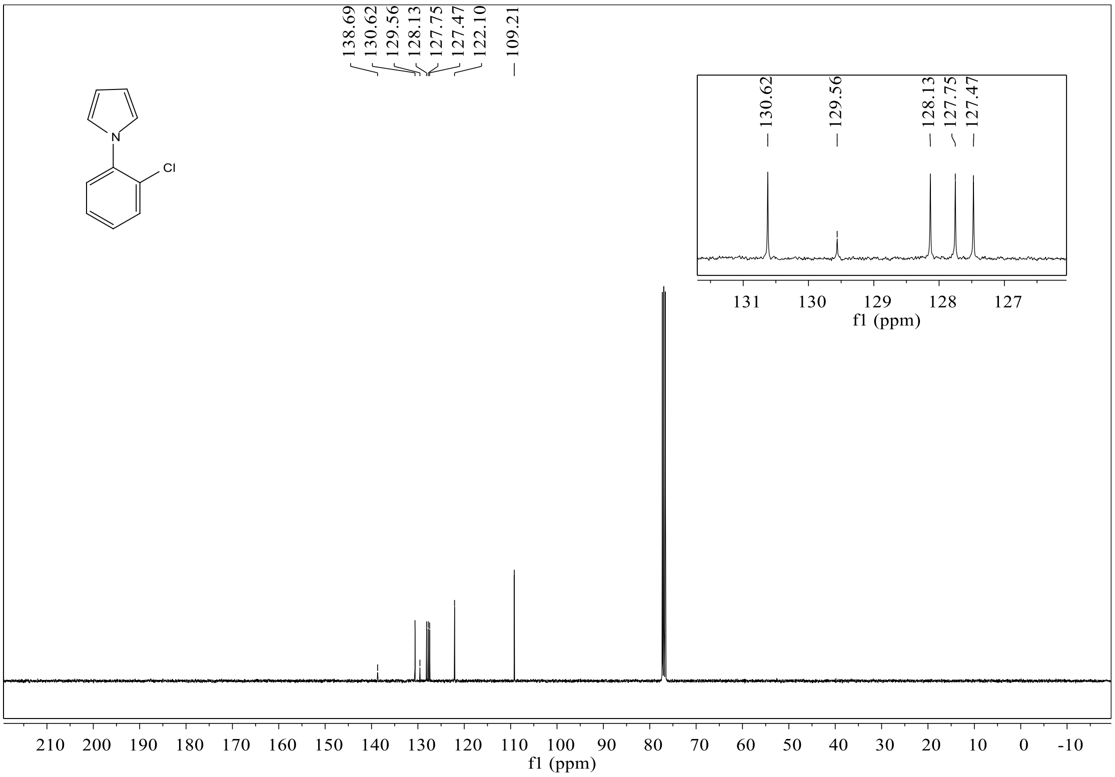

^{1}H NMR spectrum of compound 3c (400 MHz, $CDCl_3$)

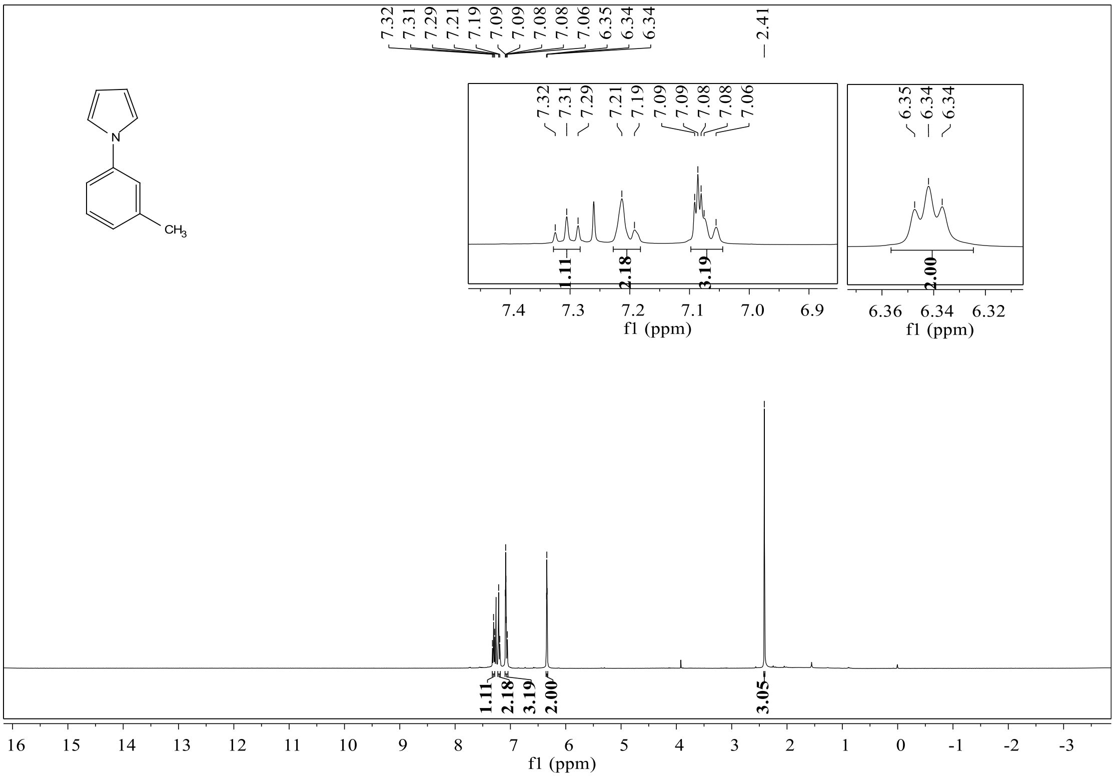

^{13}C NMR spectrum of compound 3c (76 MHz, $CDCl_3$)

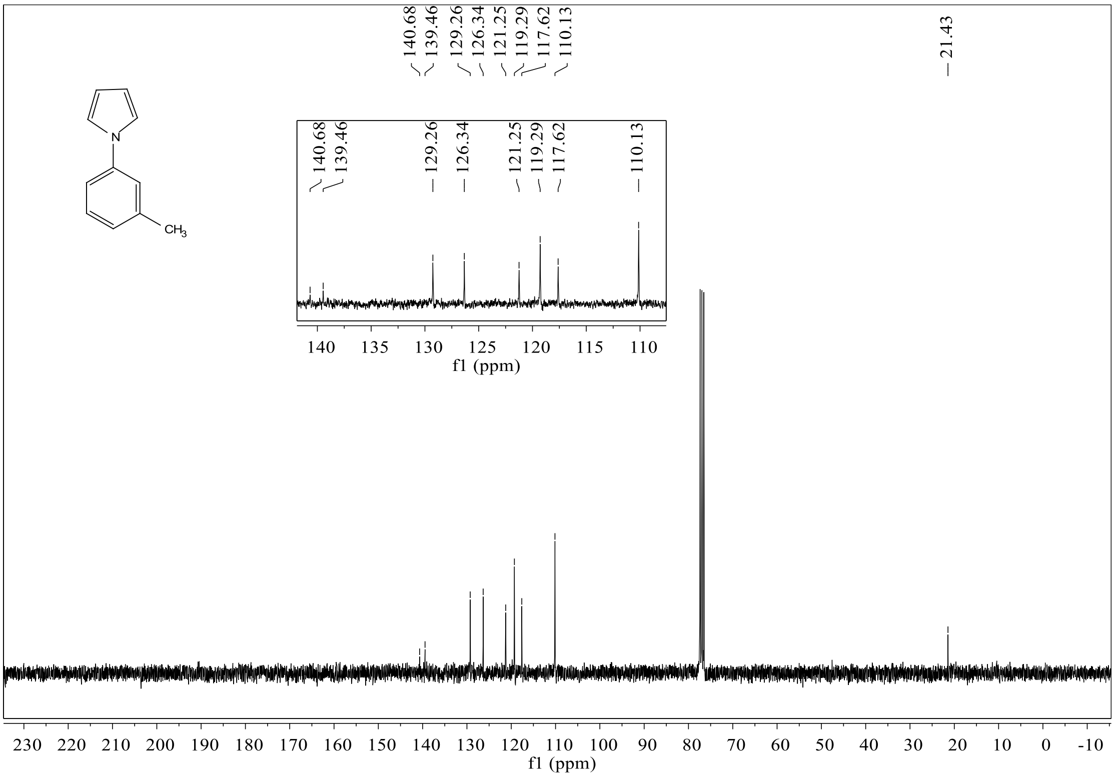

^{1}H NMR spectrum of compound 3d (400 MHz, $CDCl_3$)

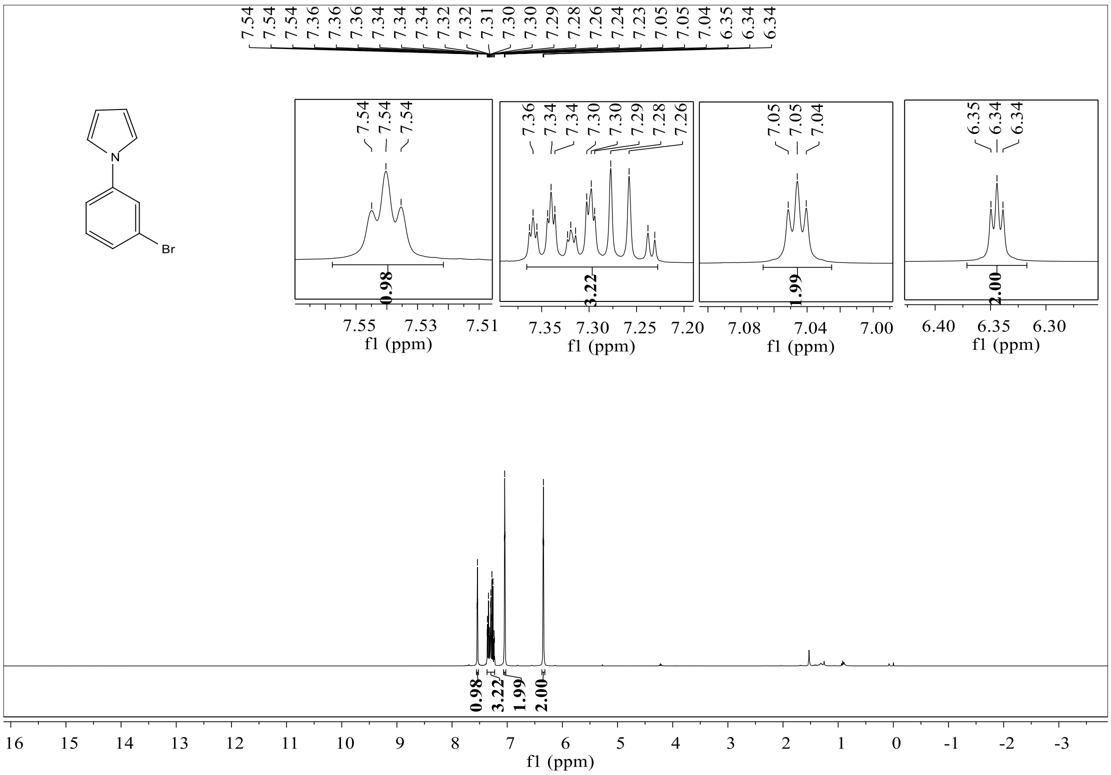

^{13}C NMR spectrum of compound 3d (101 MHz, $CDCl_3$)

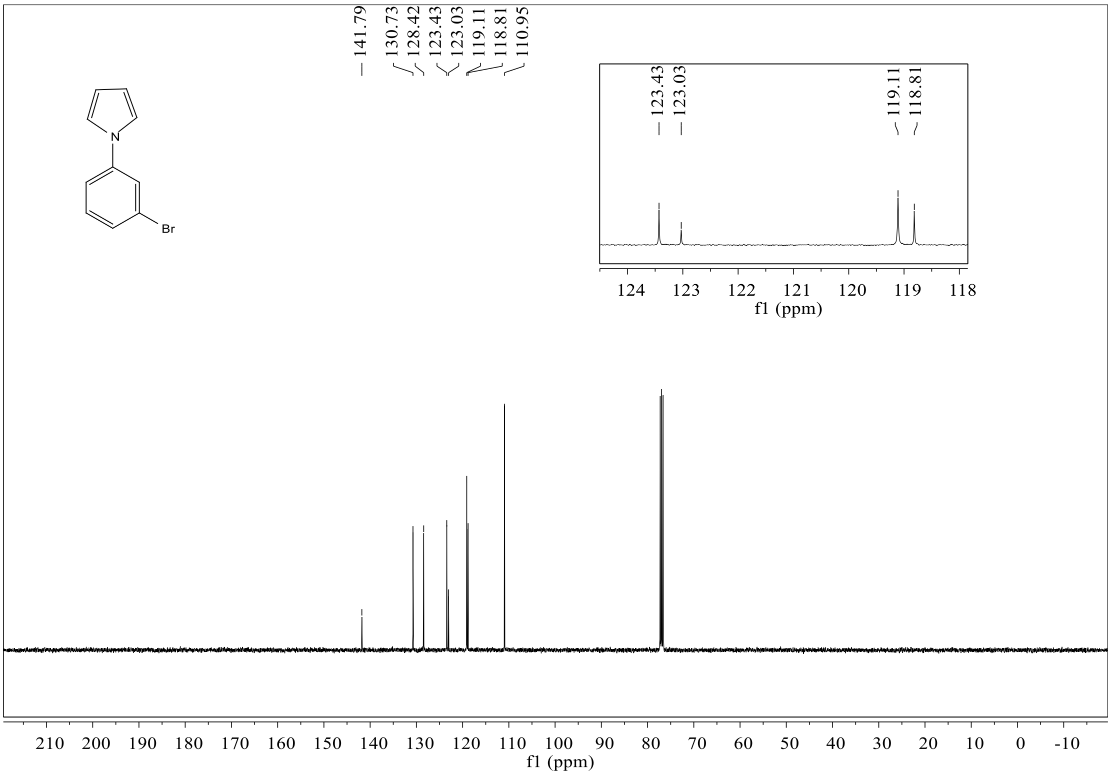

^{1}H NMR spectrum of compound 3e (400 MHz, $CDCl_3$)

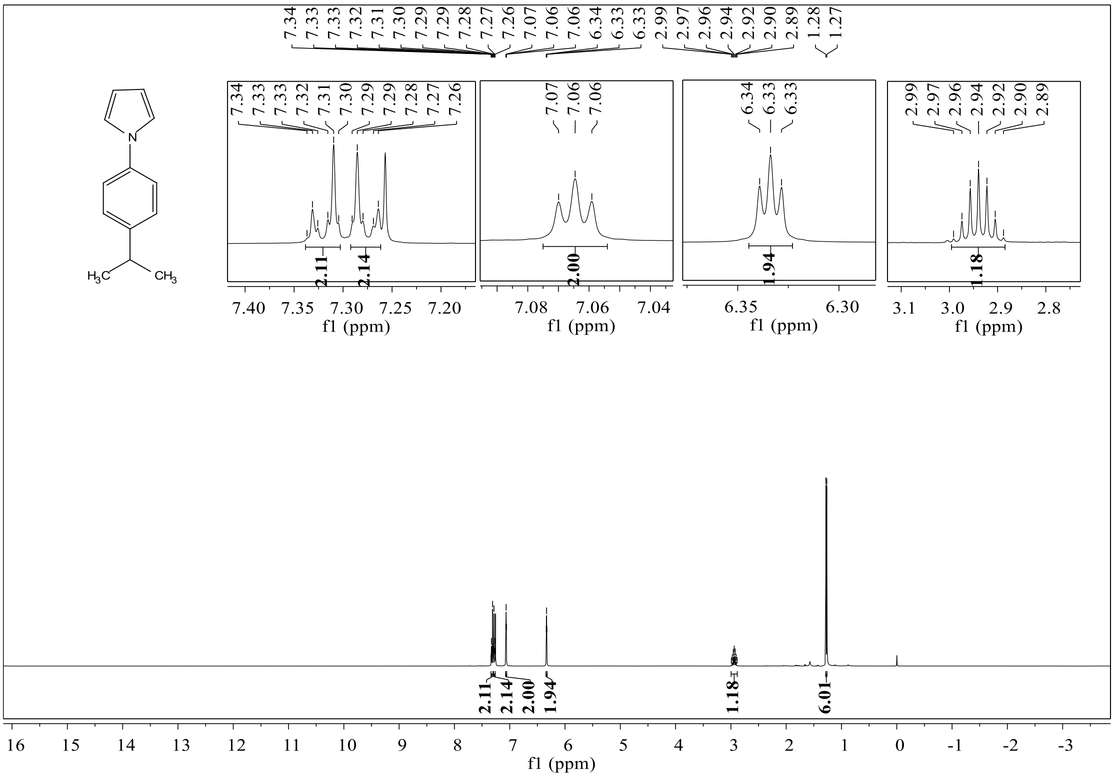

^{13}C NMR spectrum of compound 3e (101 MHz, $CDCl_3$)

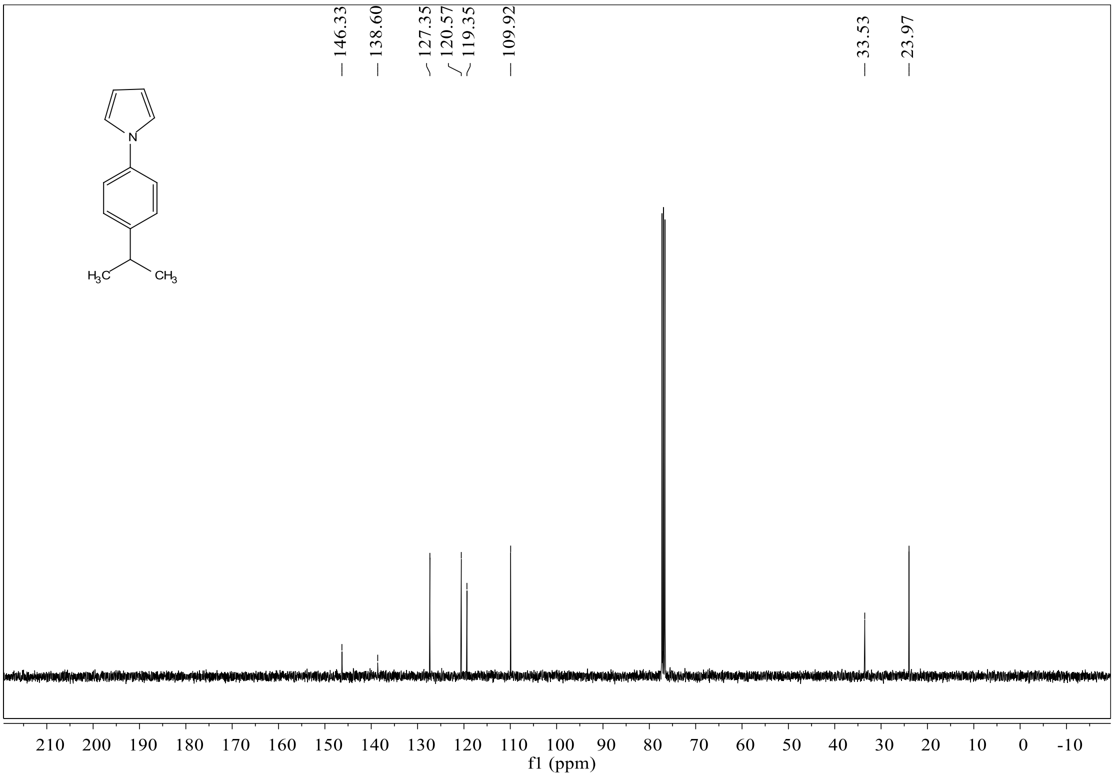

^{1}H NMR spectrum of compound 3f (400 MHz, $CDCl_3$)

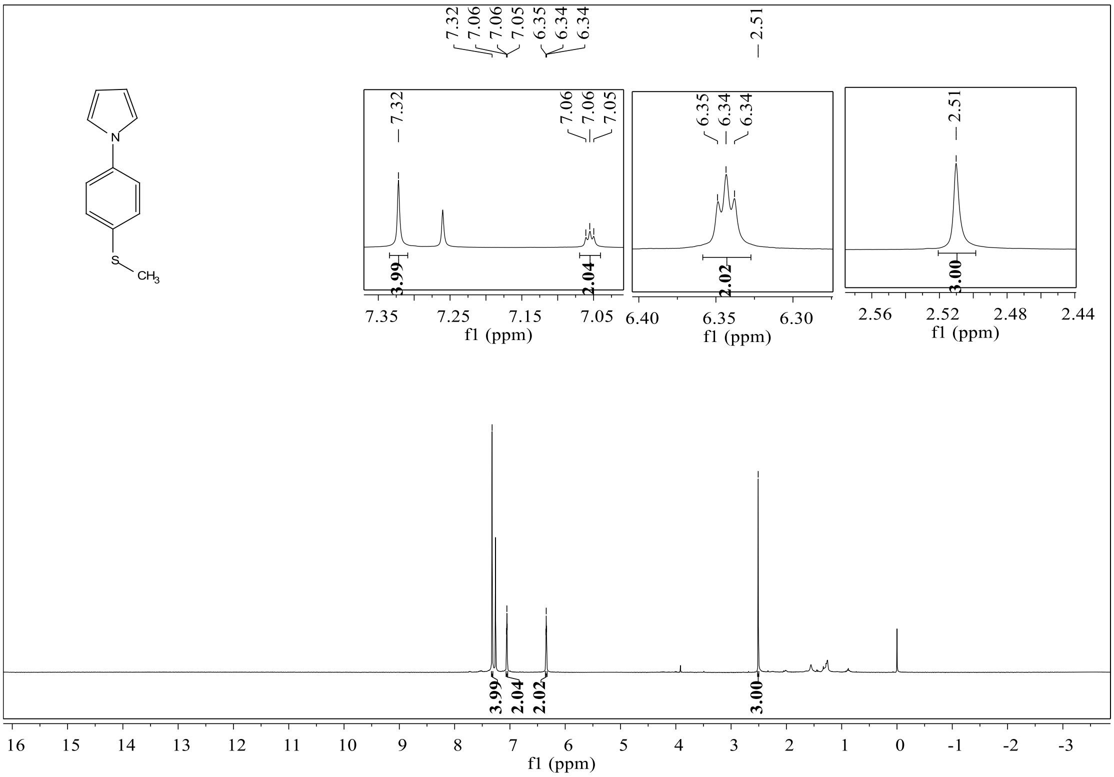

^{13}C NMR spectrum of compound 3f (101 MHz, $CDCl_3$)

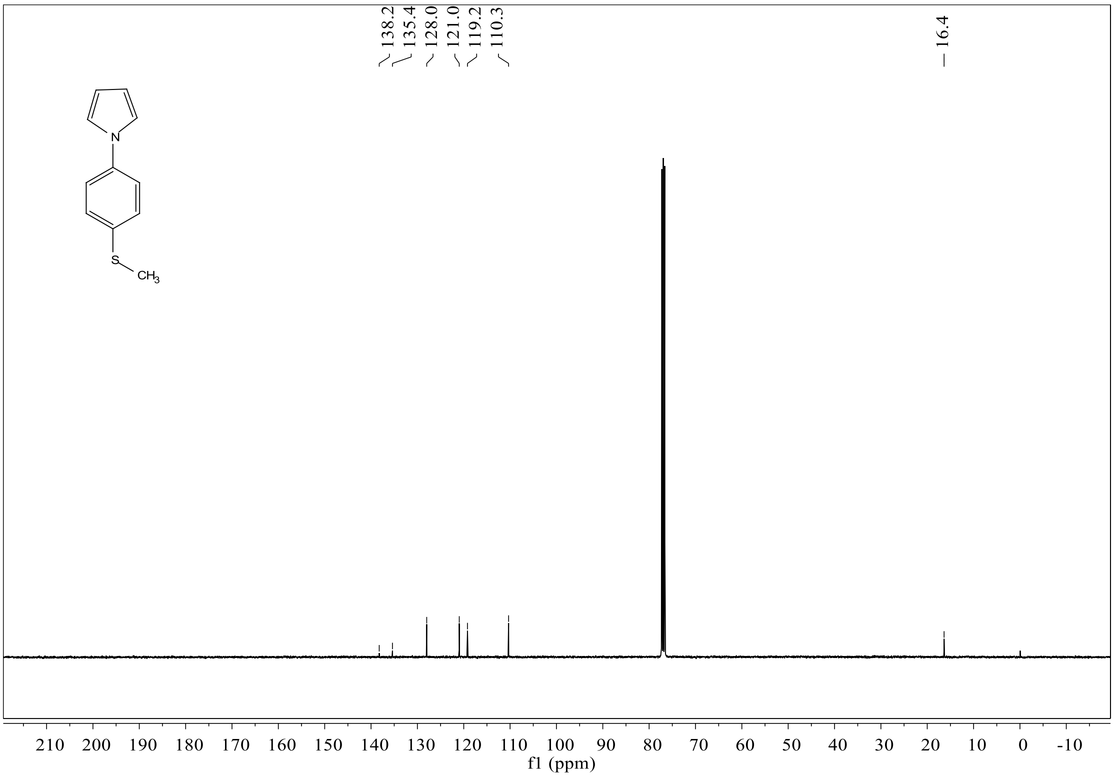

^1H NMR spectrum of compound 3g (400 MHz, $CDCl_3$)

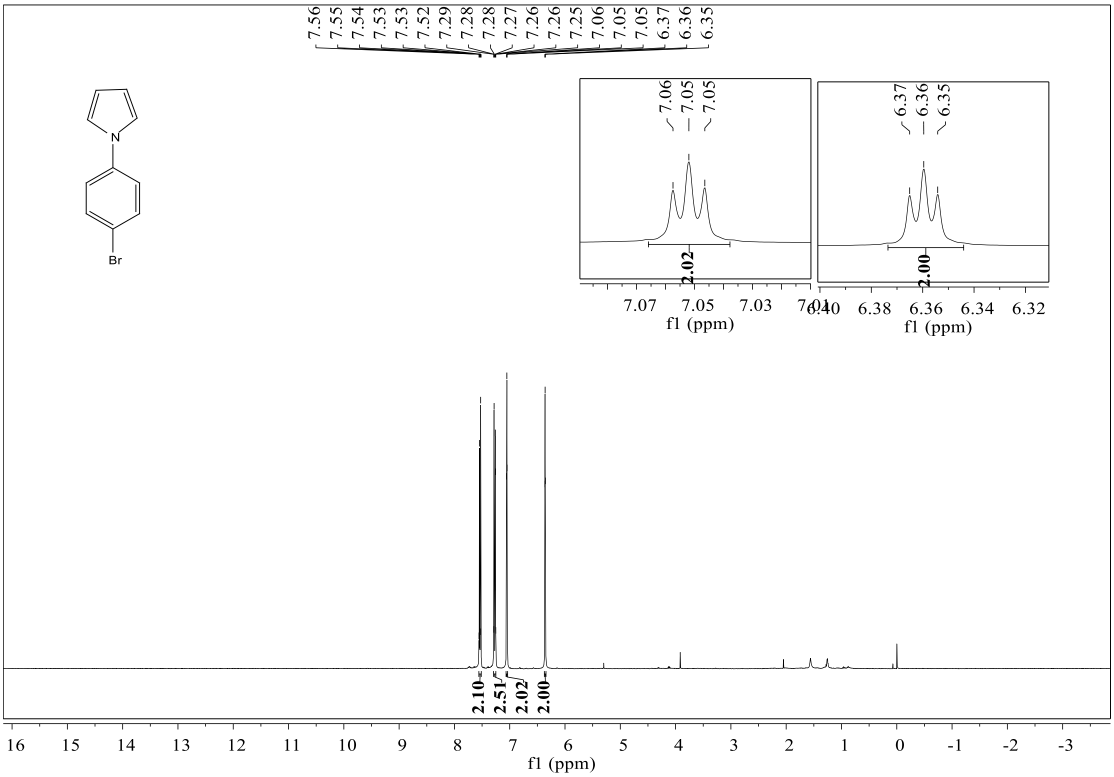

^{13}C NMR spectrum of compound 3g (101 MHz, $CDCl_3$)

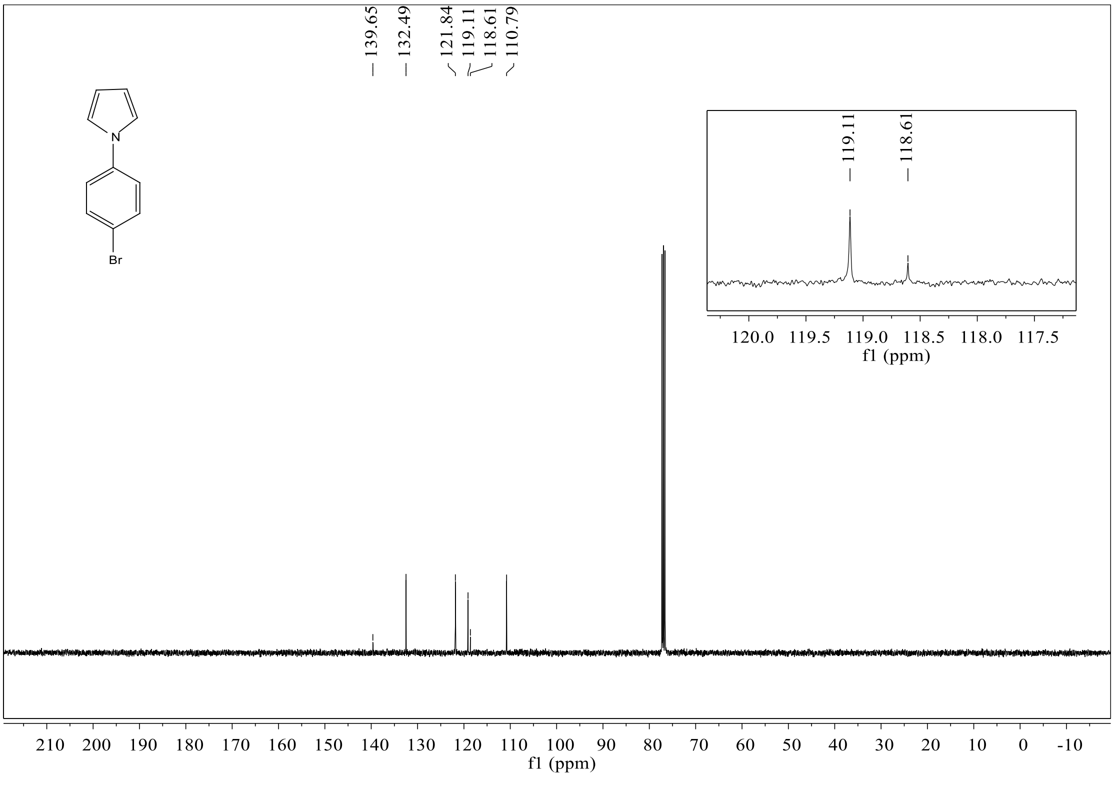

^{1}H NMR spectrum of compound 3h (400 MHz, $CDCl_3$)

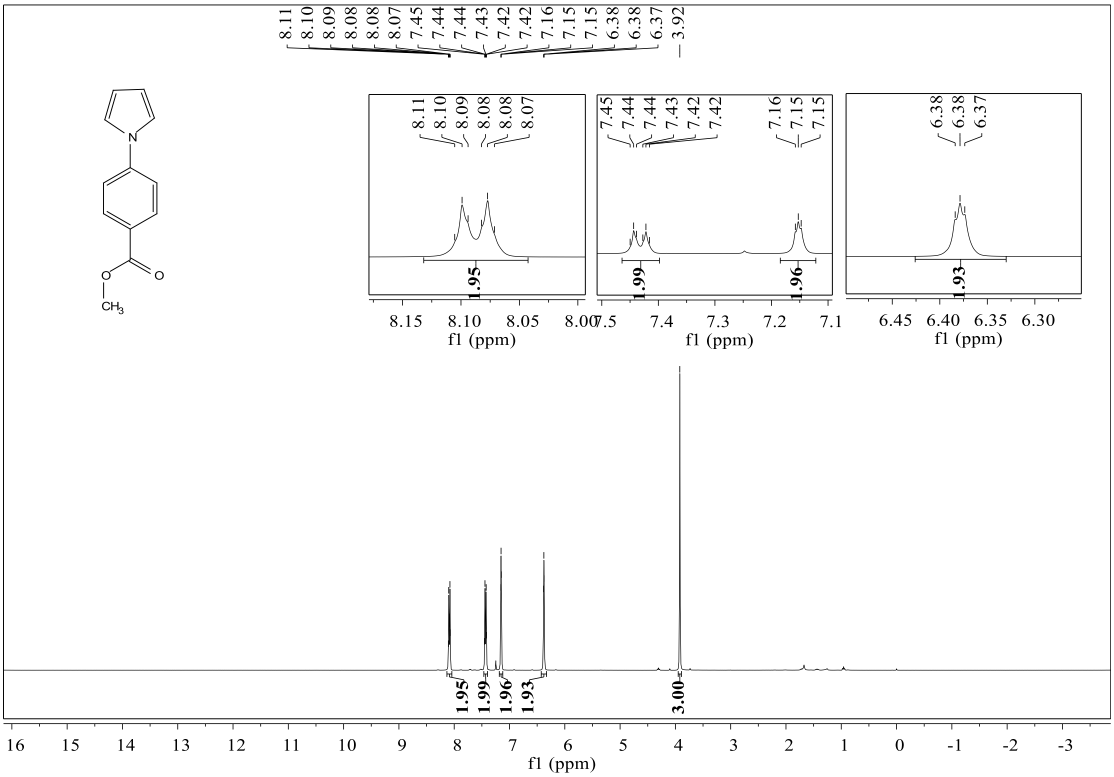

^{13}C NMR spectrum of compound 3h (101 MHz, $CDCl_3$)

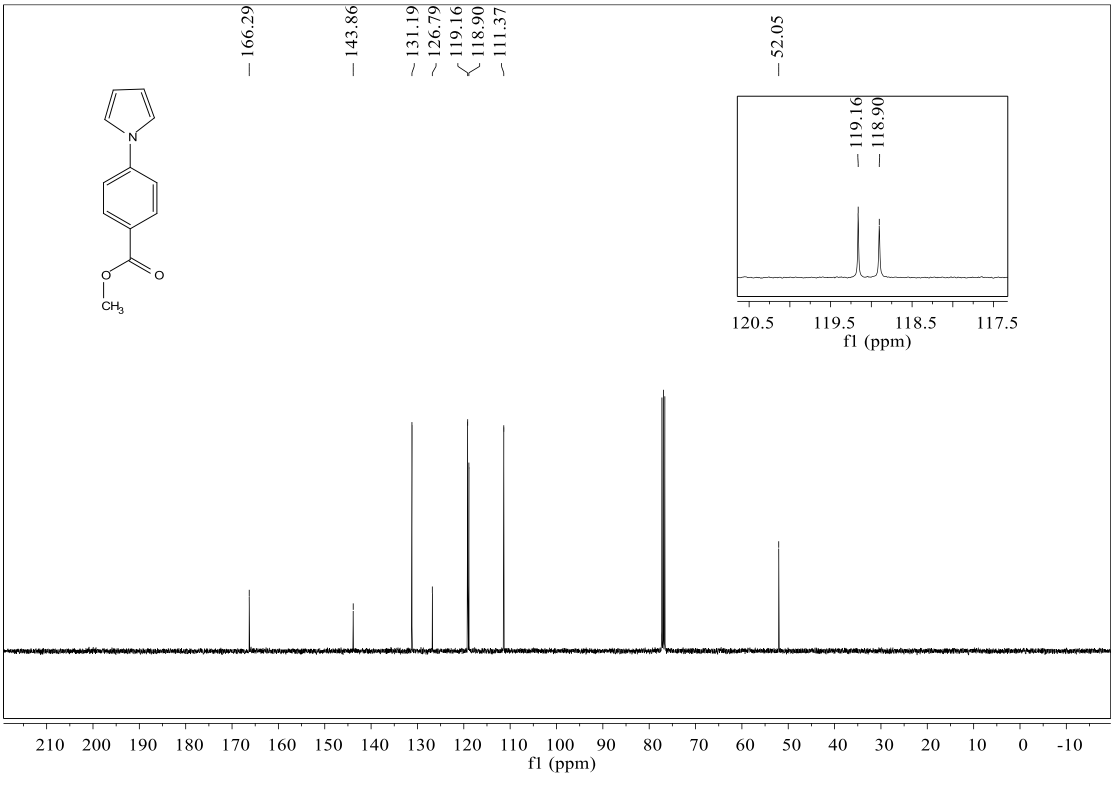

^{1}H NMR spectrum of compound 3i (400 MHz, $CDCl_3$)

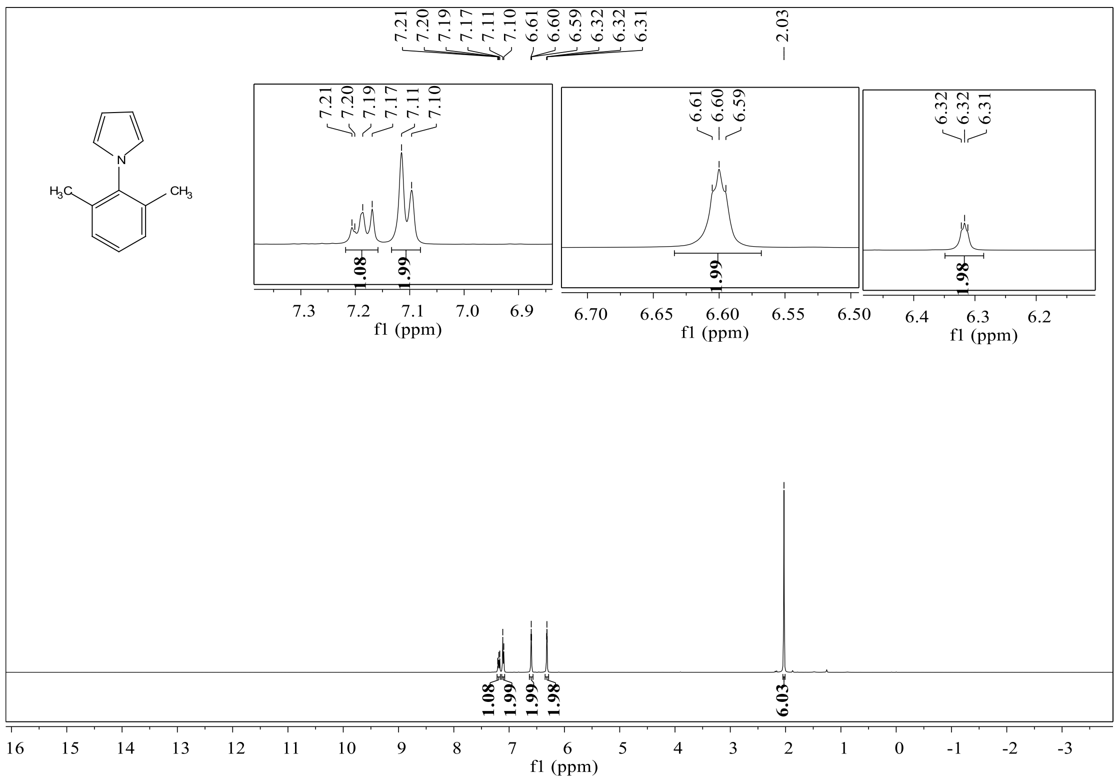

^{13}C NMR spectrum of compound 3i (101 MHz, $CDCl_3$)

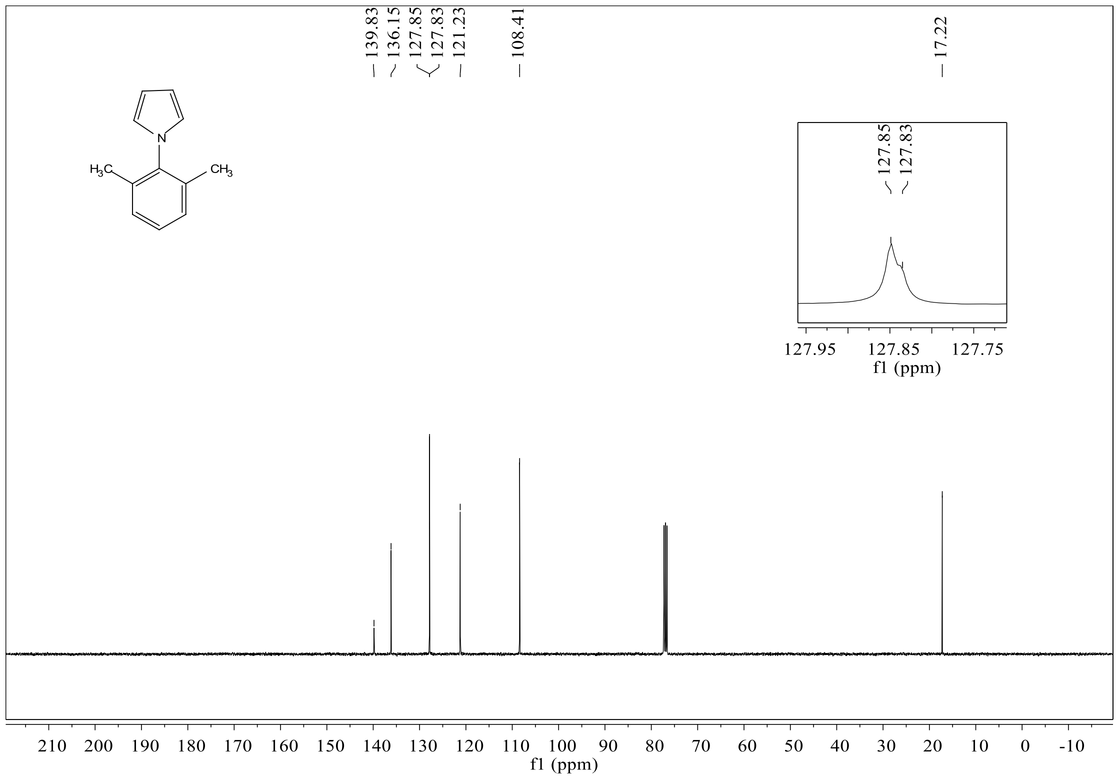

^{1}H NMR spectrum of compound 3j (400 MHz, $CDCl_3$)

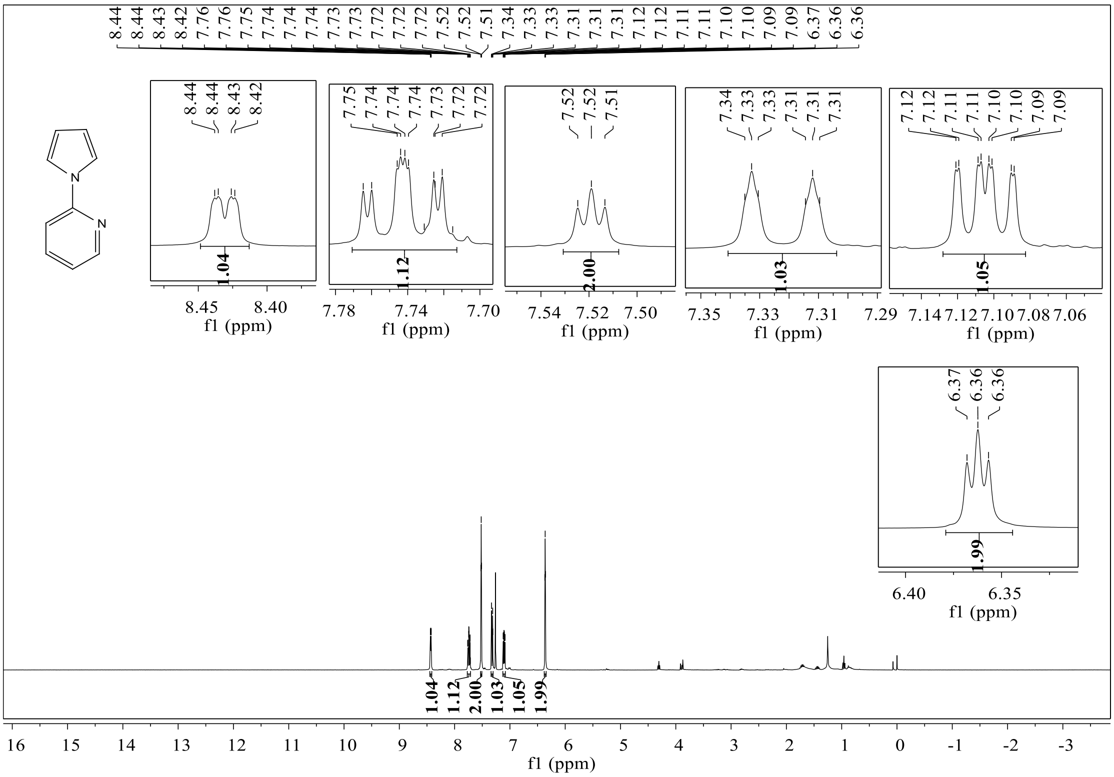

^{13}C NMR spectrum of compound 3j (101 MHz, $CDCl_3$)

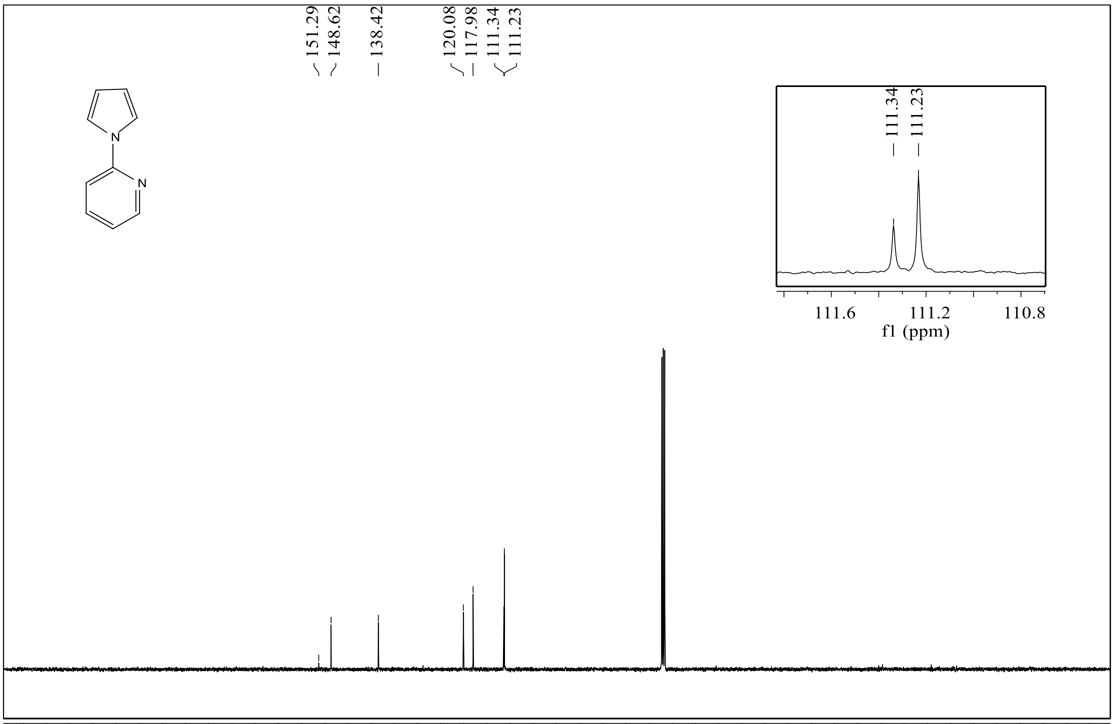

^{1}H NMR spectrum of compound 3k (400 MHz, $CDCl_3$)

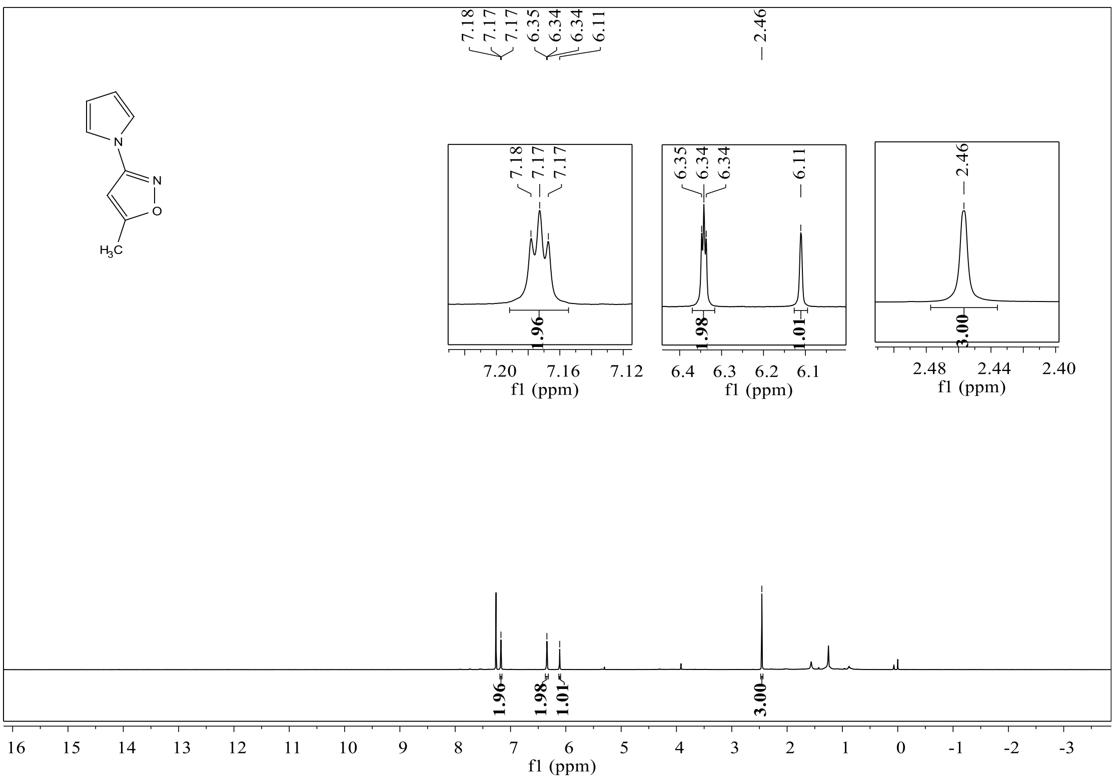

^{13}C NMR spectrum of compound 3k (101 MHz, $CDCl_3$)

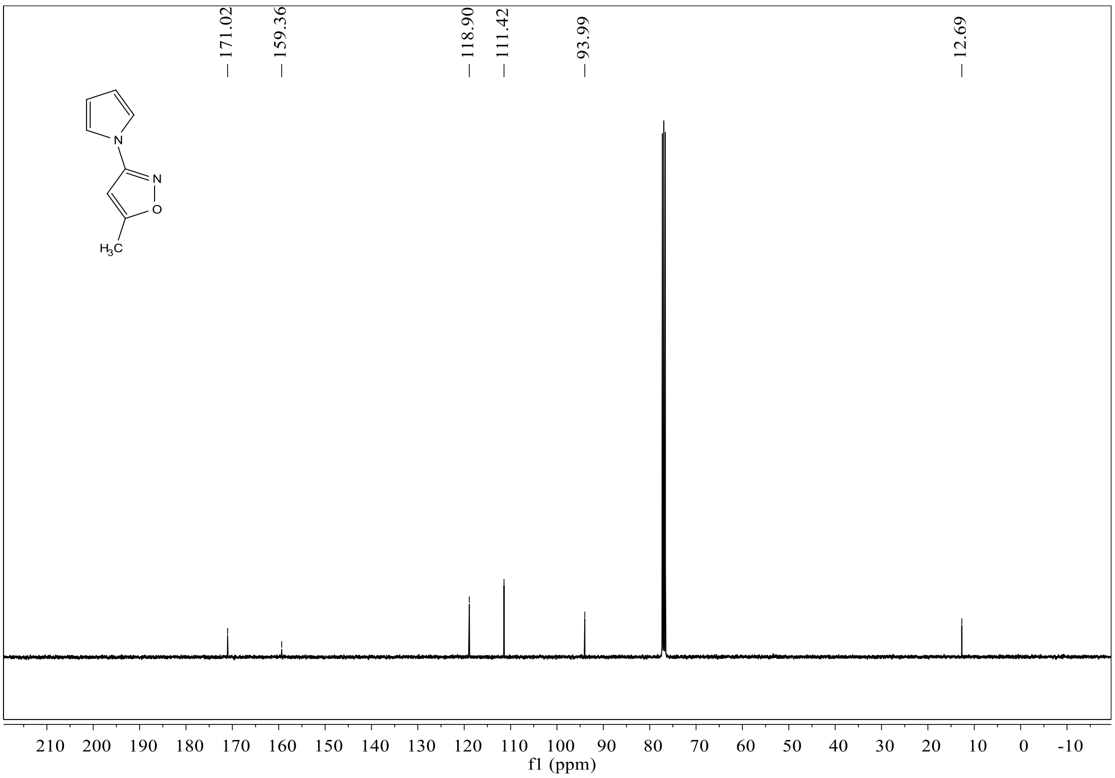

^{1}H NMR spectrum of compound 3l (400 MHz, $CDCl_3$)

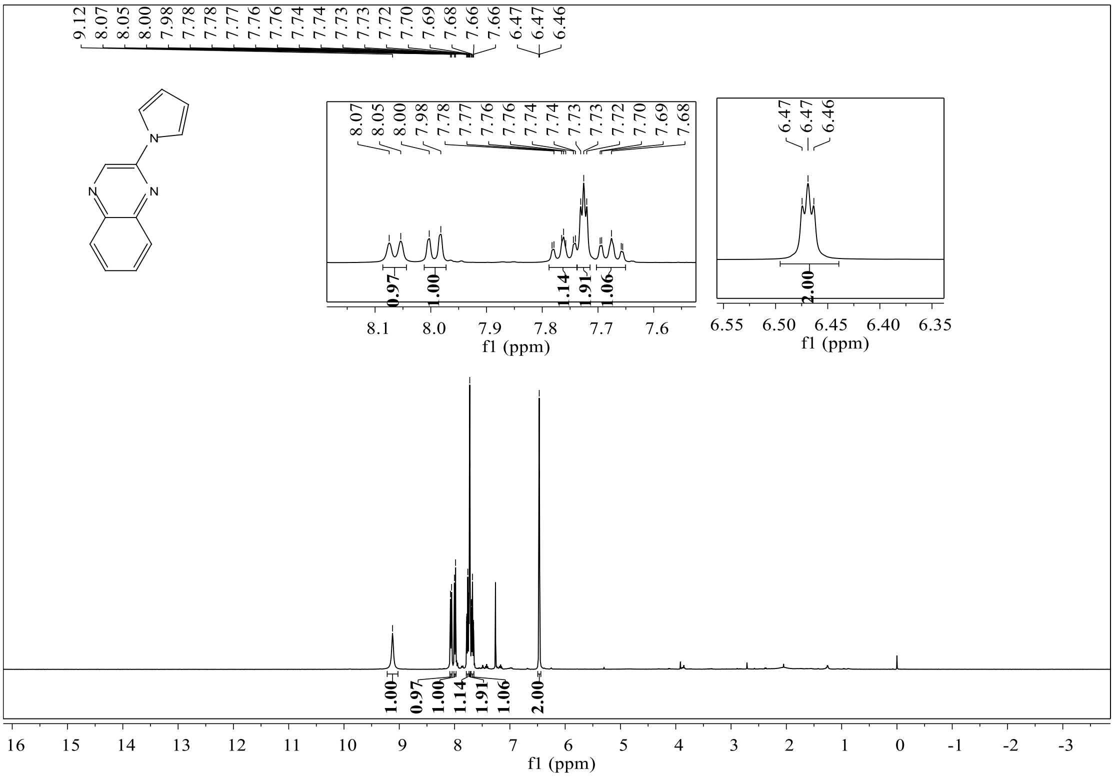

^{13}C NMR spectrum of compound 3l (101 MHz, $CDCl_3$)

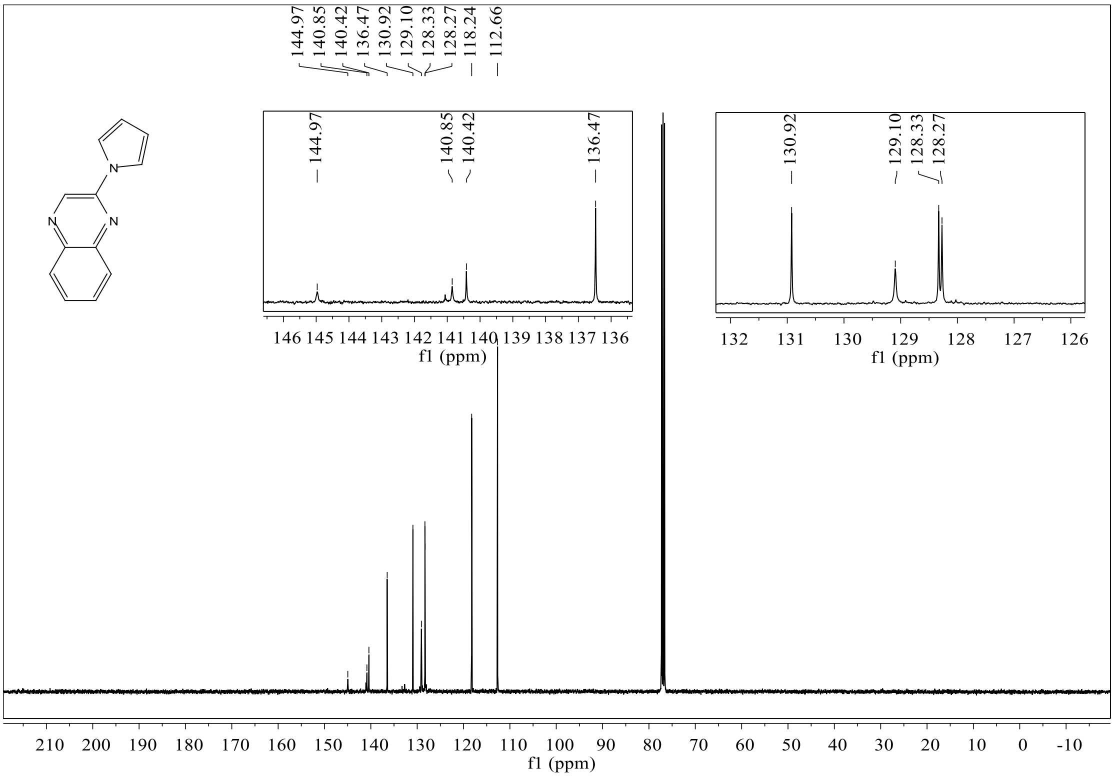

^{1}H NMR spectrum of compound 3m (400 MHz, $CDCl_3$)

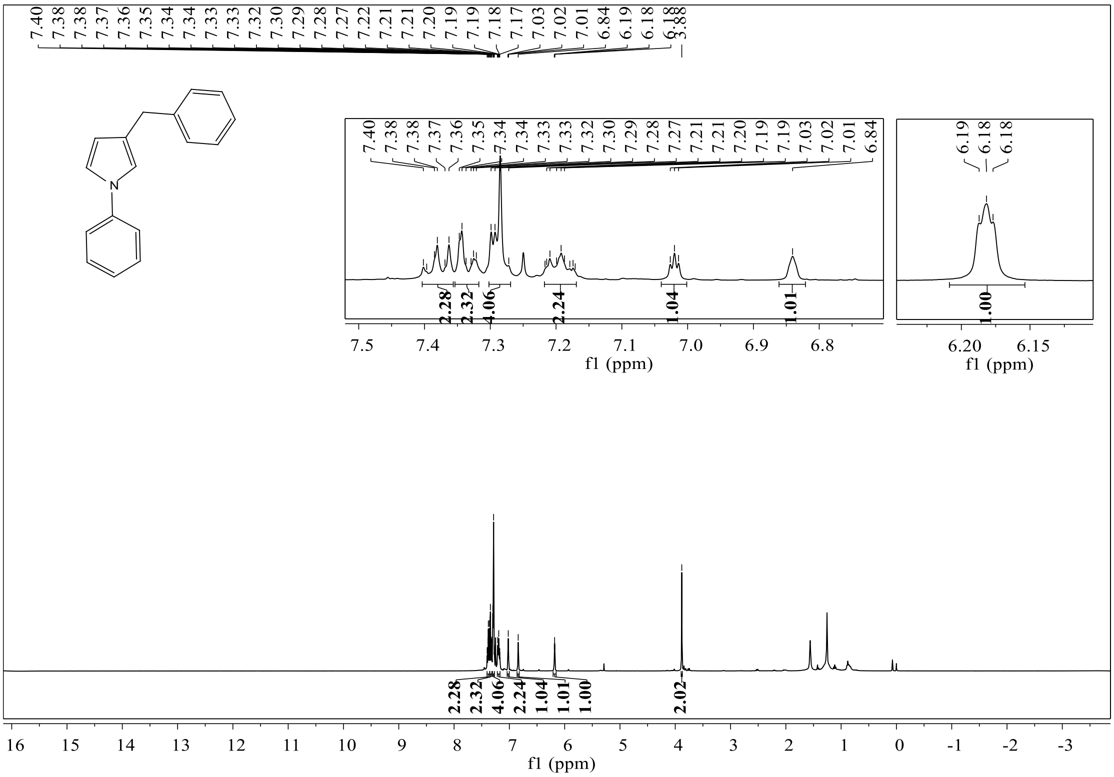

^{13}C NMR spectrum of compound 3m (101 MHz, $CDCl_3$)

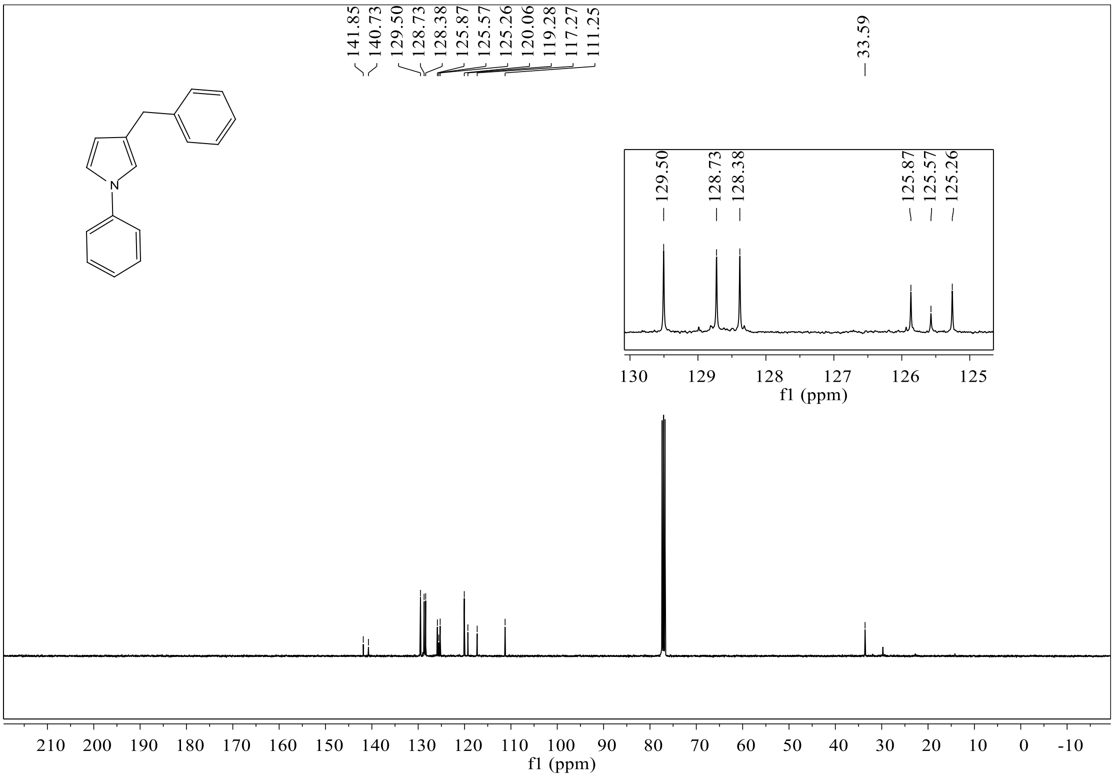

^{1}H NMR spectrum of compound 3n (400 MHz, $CDCl_3$)

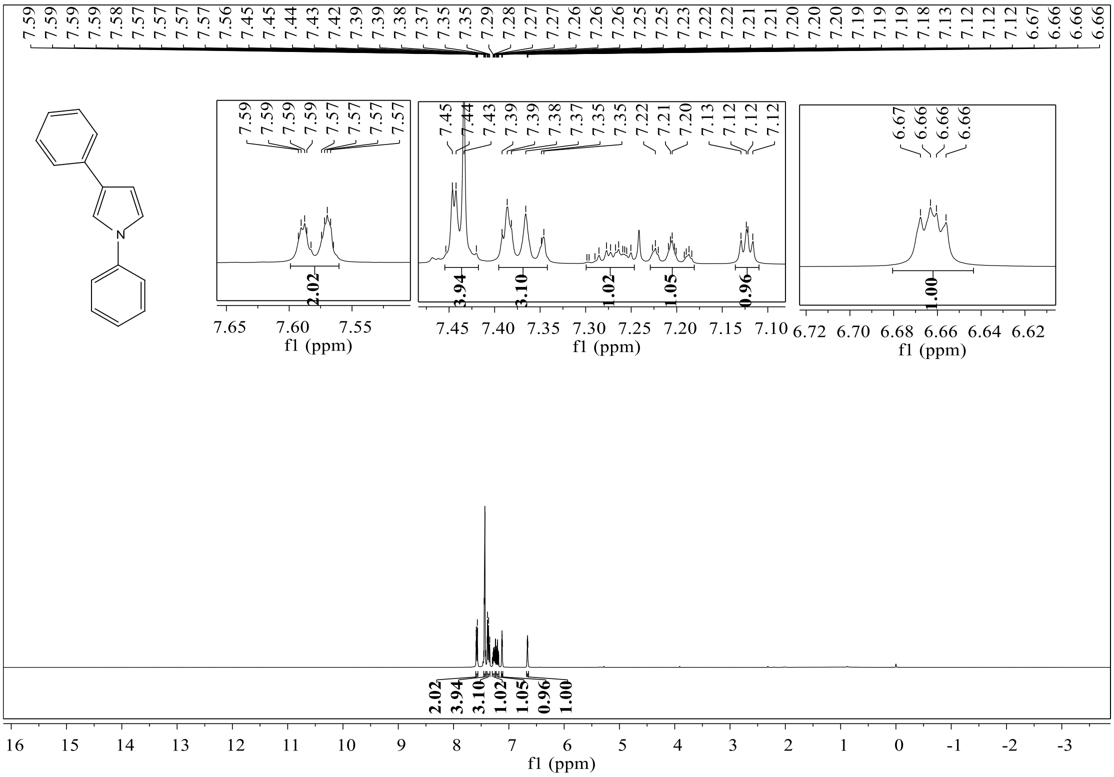

^{13}C NMR spectrum of compound 3n (101 MHz, $CDCl_3$)

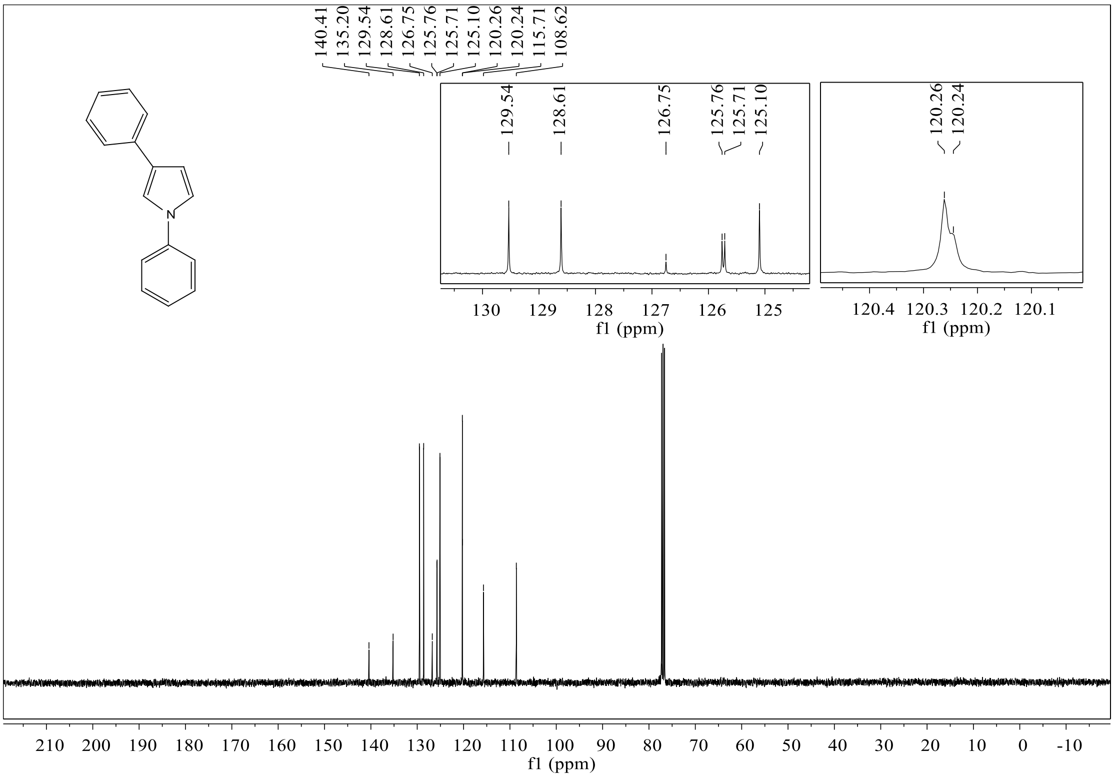

^{1}H NMR spectrum of compound 3o (400 MHz, $CDCl_3$)

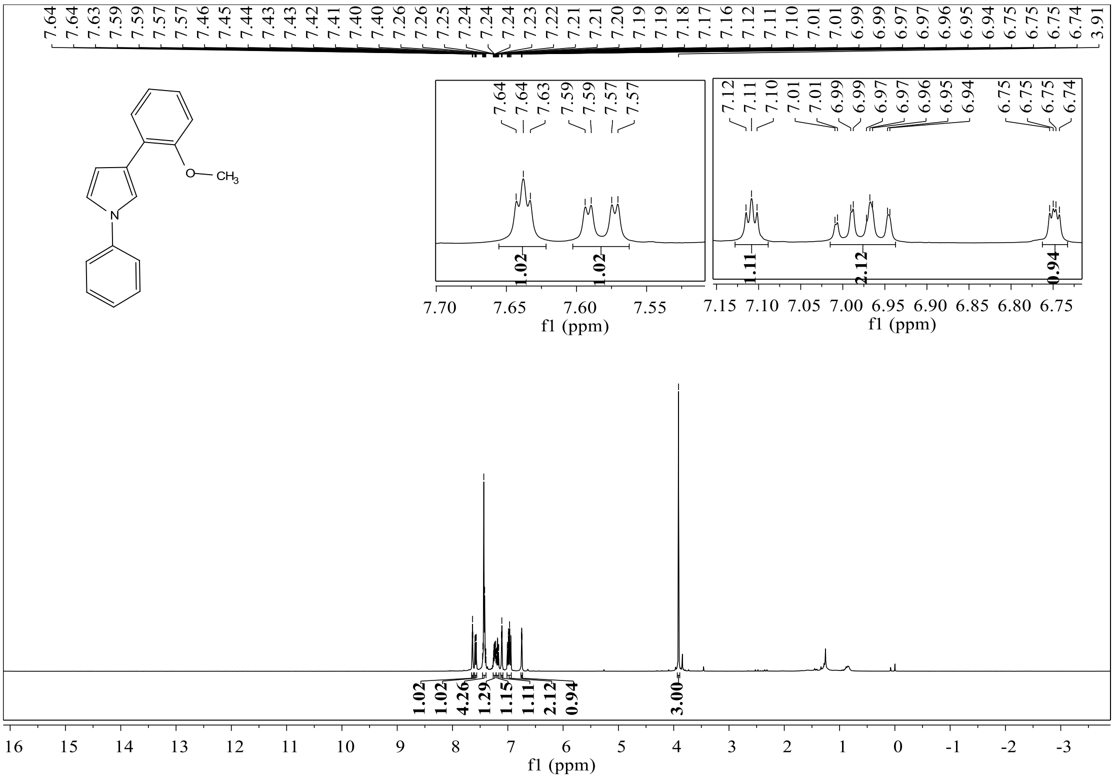

^{13}C NMR spectrum of compound 3o (101 MHz, $CDCl_3$)

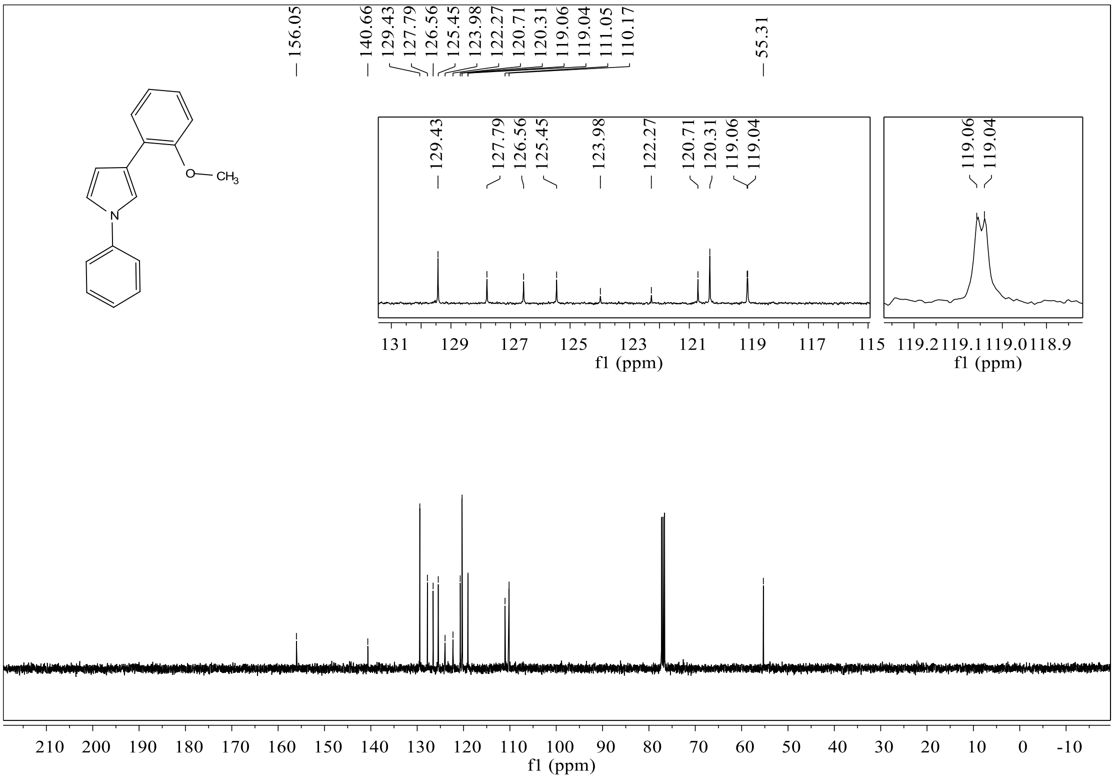

^{1}H NMR spectrum of compound 3p (400 MHz, $CDCl_3$)

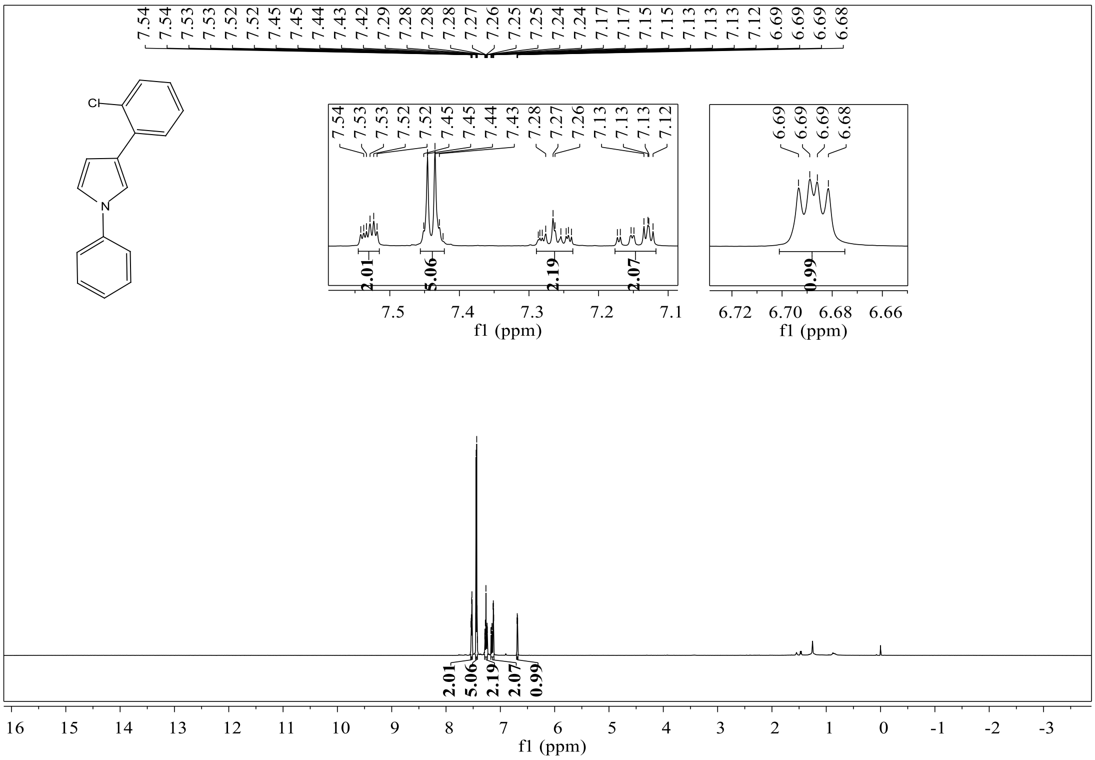

^{13}C NMR spectrum of compound 3p (101 MHz, $CDCl_3$)

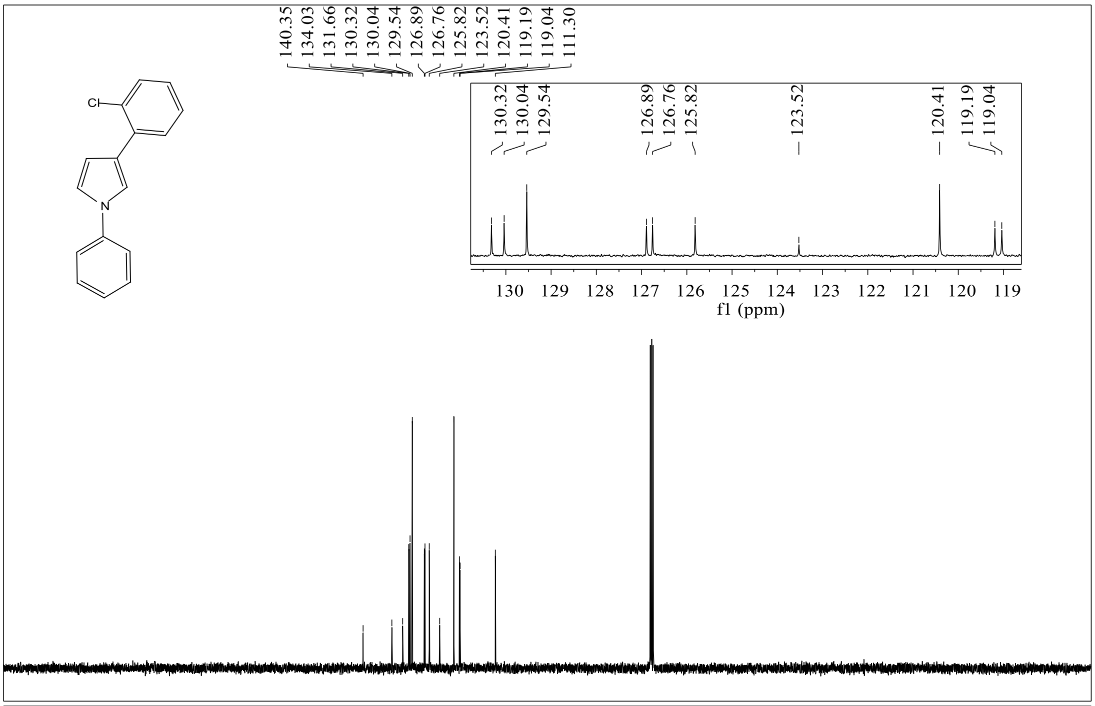

^{1}H NMR spectrum of compound 3q (400 MHz, $CDCl_3$)

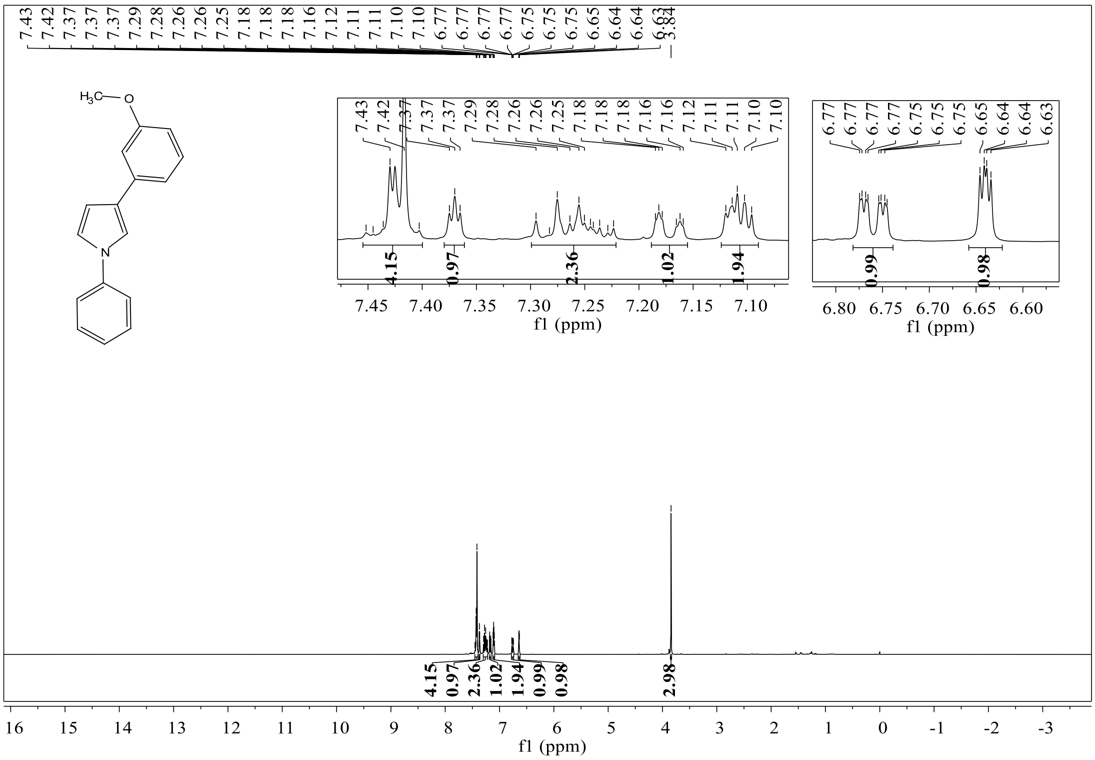

^{13}C NMR spectrum of compound 3q (101 MHz, $CDCl_3$)

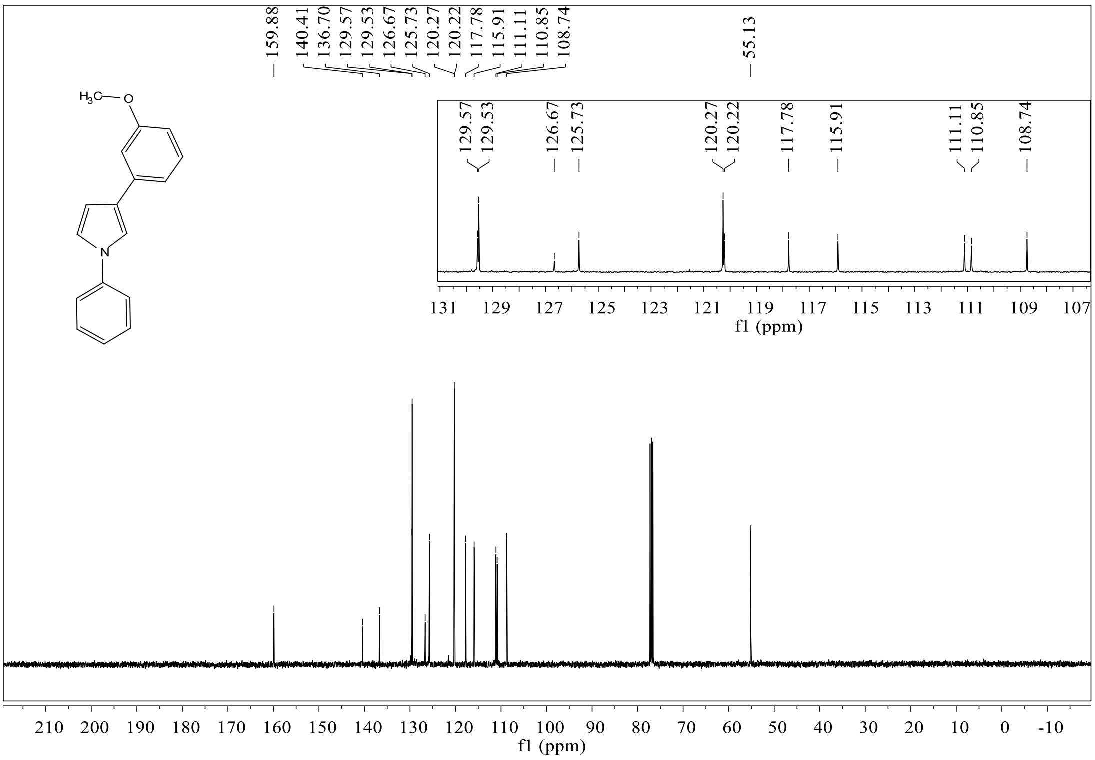

^{1}H NMR spectrum of compound 3r (400 MHz, $CDCl_3$)

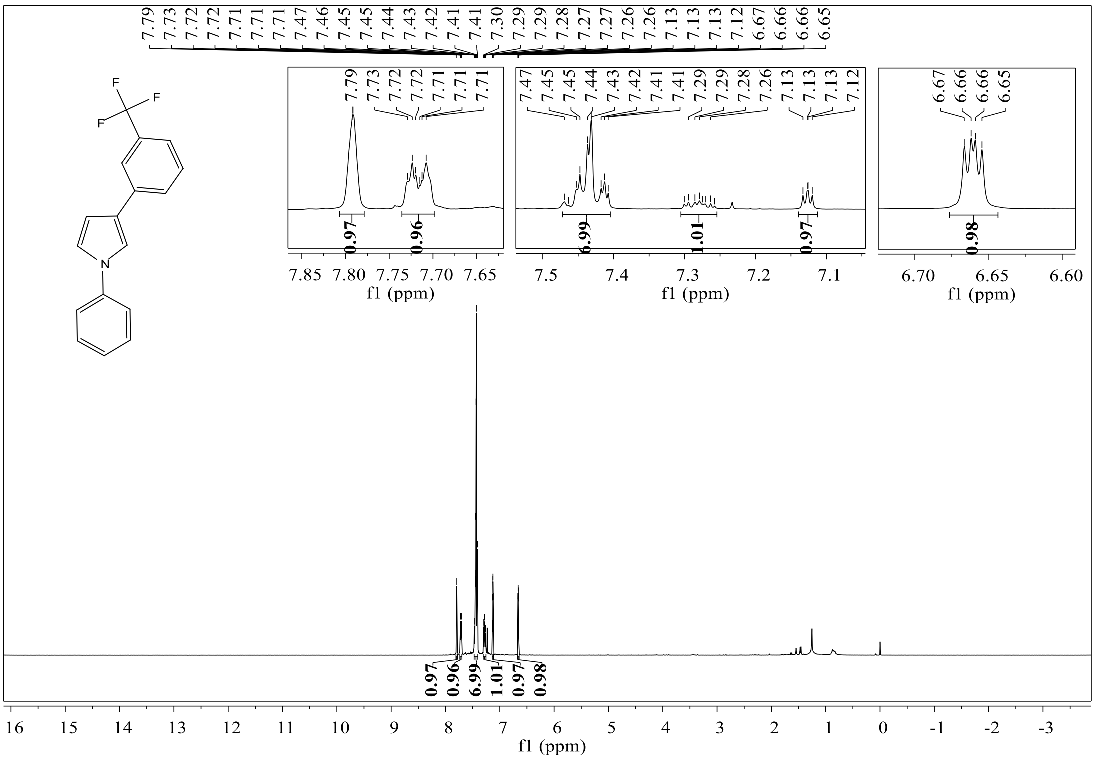

^{13}C NMR spectrum of compound 3r (101 MHz, $CDCl_3$)

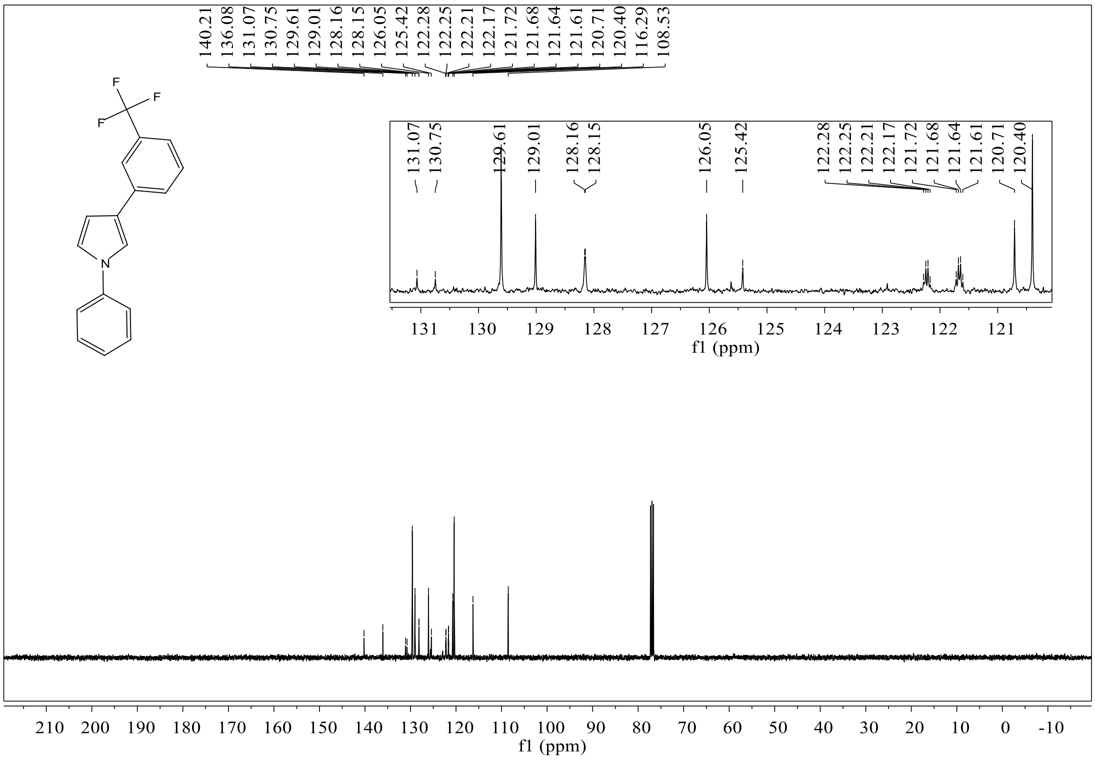

^{1}H NMR spectrum of compound 3s (400 MHz, $CDCl_3$)

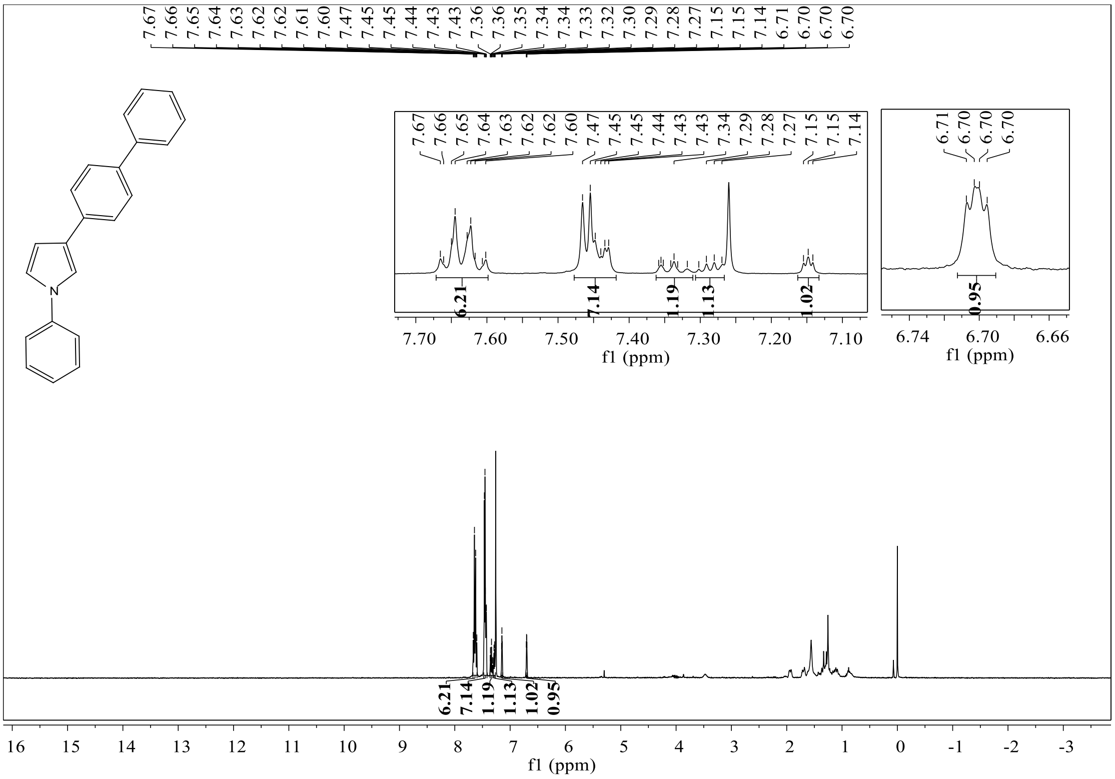

^{13}C NMR spectrum of compound 3s (101 MHz, $CDCl_3$)

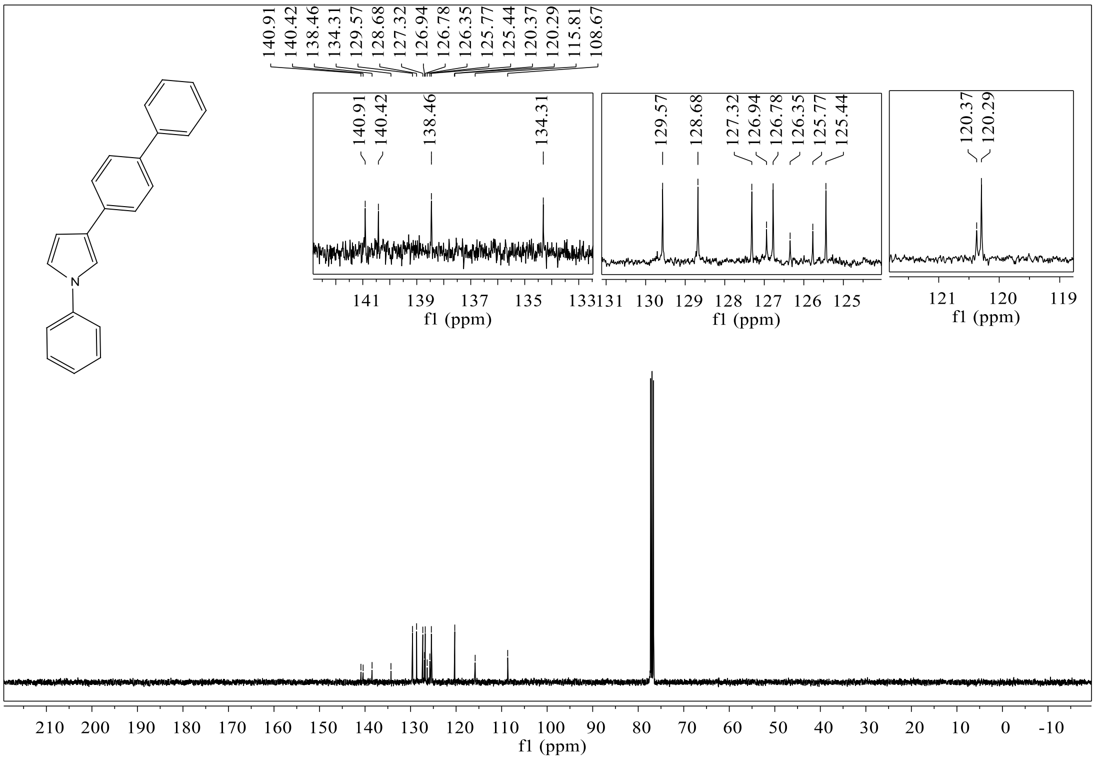

Supplement: Supplementary file 1 [file DataSheet1.pdf]
